# The longitudinal characterization of immune responses in COVID-19 patients reveals novel prognostic signatures for disease severity, patients’ survival and long COVID

**DOI:** 10.3389/fimmu.2024.1381091

**Published:** 2024-07-29

**Authors:** Maddalena Noviello, Rebecca De Lorenzo, Raniero Chimienti, Norma Maugeri, Claudia De Lalla, Gabriel Siracusano, Nicola Ivan Lorè, Paola Maria Vittoria Rancoita, Federica Cugnata, Elena Tassi, Stefania Dispinseri, Danilo Abbati, Valeria Beretta, Eliana Ruggiero, Francesco Manfredi, Aurora Merolla, Elisa Cantarelli, Cristina Tresoldi, Claudia Pastori, Roberta Caccia, Francesca Sironi, Ilaria Marzinotto, Fabio Saliu, Silvia Ghezzi, Vito Lampasona, Elisa Vicenzi, Paola Cinque, Angelo Andrea Manfredi, Gabriella Scarlatti, Paolo Dellabona, Lucia Lopalco, Clelia Di Serio, Mauro Malnati, Fabio Ciceri, Patrizia Rovere-Querini, Chiara Bonini

**Affiliations:** ^1^ Experimental Hematology Unit, Istituto di Ricovero e Cura a Carattere Scientifico (IRCCS) San Raffaele Scientific Institute, Milan, Italy; ^2^ Cell Therapy Immunomonitoring Laboratory Monitoraggio Immunologico Terapie Cellulari (MITiCi), Istituto di Ricovero e Cura a Carattere Scientifico (IRCCS) San Raffaele Scientific Institute, Milan, Italy; ^3^ Vita-Salute San Raffaele University, Milan, Italy; ^4^ Internal Medicine Unit, Istituto di Ricovero e Cura a Carattere Scientifico (IRCCS) San Raffaele Scientific Institute, Milan, Italy; ^5^ Viral Evolution and Transmission Unit, Istituto di Ricovero e Cura a Carattere Scientifico (IRCCS) San Raffaele Scientific Institute, Milan, Italy; ^6^ Autoimmunity and Vascular Inflammation Unit, Istituto di Ricovero e Cura a Carattere Scientifico (IRCCS) San Raffaele Scientific Institute, Milan, Italy; ^7^ Experimental Immunology Unit, Istituto di Ricovero e Cura a Carattere Scientifico (IRCCS) San Raffaele Scientific Institute, Milan, Italy; ^8^ Immunobiology of Human Immunodeficiency Virus (HIV) Unit, Istituto di Ricovero e Cura a Carattere Scientifico (IRCCS) San Raffaele Scientific Institute, Milan, Italy; ^9^ Emerging Bacterial Pathogens Unit, Istituto di Ricovero e Cura a Carattere Scientifico (IRCCS) San Raffaele Scientific Institute, Milan, Italy; ^10^ University Centre for Statistics in the Biomedical Sciences (CUSSB), Vita-Salute San Raffaele University, Milan, Italy; ^11^ Biological Resource Center Centro Risorse Biologiche-Ospedale San Raffaele (CRB-OSR), Istituto di Ricovero e Cura a Carattere Scientifico (IRCCS) San Raffaele Scientific Institute, Milan, Italy; ^12^ Neurovirology Unit, Istituto di Ricovero e Cura a Carattere Scientifico (IRCCS) San Raffaele Scientific Institute, Milan, Italy; ^13^ Diabetes Research Institute, Istituto di Ricovero e Cura a Carattere Scientifico (IRCCS) Ospedale San Raffaele, Milan, Italy; ^14^ Viral Pathogenesis and Biosafety Unit, Istituto di Ricovero e Cura a Carattere Scientifico (IRCCS) San Raffaele Scientific Institute, Milan, Italy; ^15^ Hematology and Bone Marrow Transplant Unit, Istituto di Ricovero e Cura a Carattere Scientifico (IRCCS) San Raffaele Scientific Institute, Milan, Italy

**Keywords:** COVID-19 severity, COVID-19 patients’ survival, SARS-CoV-2 innate immunity, SARS-CoV-2 adaptive immunity, long COVID

## Abstract

**Introduction:**

SARS-CoV-2 pandemic still poses a significant burden on global health and economy, especially for symptoms persisting beyond the acute disease. COVID-19 manifests with various degrees of severity and the identification of early biomarkers capable of stratifying patient based on risk of progression could allow tailored treatments.

**Methods:**

We longitudinally analyzed 67 patients, classified according to a WHO ordinal scale as having Mild, Moderate, or Severe COVID-19. Peripheral blood samples were prospectively collected at hospital admission and during a 6-month follow-up after discharge. Several subsets and markers of the innate and adaptive immunity were monitored as putative factors associated with COVID-19 symptoms.

**Results:**

More than 50 immunological parameters were associated with disease severity. A decision tree including the main clinical, laboratory, and biological variables at admission identified low NK-cell precursors and CD14^+^CD91^+^ monocytes, and high CD8^+^ Effector Memory T cell frequencies as the most robust immunological correlates of COVID-19 severity and reduced survival. Moreover, low regulatory B-cell frequency at one month was associated with the susceptibility to develop long COVID at six months, likely due to their immunomodulatory ability.

**Discussion:**

These results highlight the profound perturbation of the immune response during COVID-19. The evaluation of specific innate and adaptive immune-cell subsets allows to distinguish between different acute and persistent COVID-19 symptoms.

## 1 Introduction

The Coronavirus disease 2019 (COVID-19) pandemic, which was declared a Public Health Emergency of International Concern by the WHO in 2020, continues to pose a substantial burden on global health and economy, with clinical manifestations often persisting beyond the acute phase of the disease (WHO Coronavirus (COVID-19) Dashboard, https://covid19.who.int).

Vaccination strategies were game-changers in fighting severe acute respiratory syndrome coronavirus 2 (SARS-CoV-2) infection. However, the continuous emergence of variants that can evade the immune response induced by vaccines still represents a threat (1, 2). Moreover, even when vaccinated, fragile individuals develop lower humoral responses than healthy subjects (3, 4) and are at risk of developing more severe symptoms upon infection. Also, in low-income countries, where vaccine accessibility remains limited (5), the vaccination rate is still insufficient to limit the spreading of the virus and the emergence of new variants.

COVID-19 spans a wide spectrum of disease severity, ranging from mild to moderate, to severe. Several therapies have been approved for the treatment of acute COVID-19 (https://www.covid19treatmentguidelines.nih.gov) based on disease severity. In this context, the identification of early biomarkers that can stratify patients based on the risk of progression are crucial for tailoring clinical intervention. The progression of severe acute COVID-19 is characterized by two main phases: 1) SARS-CoV-2 replication, mostly asymptomatic or associated with mild upper respiratory symptoms, and 2) a desynchronized immune activation that persists beyond viral clearance, leading to tissue damage, pneumonia and/or thrombotic events. Given the major role of immune dysregulation in the pathophysiology of COVID-19, specific immune biomarkers have been investigated (6). However, the dynamic role of different arms of the immune response to SARS-CoV-2 in the clinical evolution of COVID-19 is yet to be clarified.

SARS-CoV-2 infection can trigger Cytokine Release Syndrome (CRS), leading to Acute Respiratory Distress Syndrome (ARDS), multi-organ damage, and adverse clinical outcomes (7–9). CRS is characterized by a massive release of proinflammatory soluble mediators (cytokine storm) and an IL-6-driven expansion of myeloid populations expressing low levels of HLA-DR (10), including myeloid-derived suppressor cells, MDSC (11). The increase of MDSC frequency and impaired antigen presentation capacity in the myeloid compartment are associated with disease severity and may lead to the inhibition of other immune effector cells, such as NK cells, and B and T lymphocytes. In most severe cases, this process generates a state of immune-paralysis, as highlighted by gene expression analysis at single-cell resolution (12).

Lymphocytopenia is a common observation at hospital admission in COVID-19 patients and associates with adverse clinical outcomes (13, 14). Marked lymphocytopenia is accompanied by an unbalanced frequency of B and T cells (15, 16). During viral infections, B cells produce protective IgM and IgG antibodies that play a central role in controlling SARS-CoV-2 infection (17–19). Moreover, high titers of IgA antibodies (20) and of neutralizing antibodies (21) are independently associated with a more severe disease. Inefficient humoral responses in severely ill patients may be due to low B cell counts, B-cell dysfunction or lymph node damage, and, coupled with the cytotoxic impairment of CD8 T cells and NK cells, leads to unresolved inflammation and delayed viral clearance (22, 23). The quality of T-cell help is also important, as T-follicular helper cells, required to induce specific B-cell responses, were found to be defective in germinal centers from acutely ill patients (24).

Among T cells, SARS-CoV-2-specific CD8^+^ and CD4^+^ T cells have been recently identified in the majority of COVID-19 convalescent patients (25) and in vaccinated subjects (26). It is now recognized that a general loss of coordination between different branches of the immune system, particularly a delayed specific T-cell response, is associated to severe SARS-CoV-2 infection (27). Several factors may contribute to this delay, including the aforementioned lymphocytopenia in severe COVID-19 (28), the scarcity of naive T cells due to age-related thymic involution in elderly individuals (29), and inadequate signals during T-cell activation that may result in inefficient T-cell responses (16).

To shed light on the variability of immunological profiles associated with the disease, we herein provide a comprehensive and longitudinal analysis, from hospital admission during acute disease to six months after hospital discharge, in a cohort of hospitalized patients. This analysis includes: i) an in-depth phenotypic characterization of monocytes, NK cells, B and T lymphocytes; ii) the quantitative analysis of 18 chemokines and cytokines involved in the disease severity (30, 31); iii) the functional evaluation of SARS-CoV-2-specific T-cell and humoral responses. This combined assessment of different branches of the immune response led to the identification of several immunological correlates of severity of SARS-CoV-2 infection and clinical outcome. Such immune correlates have the potential to guide patient management, particularly for critically ill patients, and to design the development of innovative therapeutic approaches for individuals with persisting post-COVID19 manifestations.

## 2 Results

### 2.1 Clinical characteristics of patients

We recruited acutely symptomatic patients who were admitted to the emergency department in the period from February 2020 to April 2020. We longitudinally analyzed 67 patients, of whom 97% required hospitalization. Patients were classified according to a 6-point ordinal scale (32) as recommended by the World Health Organization (WHO; https://www.who.int/publications-detail/covid-19-therapeutic-trial-synopsis). Patients were categorized into three groups: “Mild” (n=22) for a score of 1–2-3, “Moderate” (n=20) for a score of 4, and “Severe” (n=25) for a score of 5–6. In detail, “Mild” category includes clinical statuses ranging from discharge to hospital admission not requiring high flow or non-invasive ventilation; “Moderate” category includes patients admitted to the hospital for non-invasive ventilation or high-flow oxygen therapy; “Severe” category includes patients transferred to the intensive care unit (ICU) or patients who died of COVID-19. This categorization aligned with various clinical parameters, including the lowest value of the ratio of arterial oxygen partial pressure (PaO_2_ in mmHg) to fractional inspired oxygen (FiO_2_ expressed as a fraction, PaO_2_/FiO_2_ 33) (**Supplementary Figure 1A**), Absolute Lymphocyte Counts (ALC, **Supplementary Figure 1B**), as well as other inflammatory biomarkers associated with COVID-19 progression such as lactate dehydrogenase (LDH) and C-reactive protein (CPR) (**Supplementary Figures 1C, D**).

The clinical characteristics of patients, segregated by disease category, are summarized in **Table 1**. Additional patient characteristics including detailed past medical history, therapy before admission, and treatment received during hospitalization are reported in **Supplementary Table 1**. The median age was comparable across different categories, whereas more males were identified in the most severe group. The majority of patients were Caucasian. It is worth noting that male sex was recognized as a risk factor for both death and ICU admission (34). Furthermore, the frequency of patients with comorbidities was also higher in severely ill patients compared to milder disease categories.

**Table 1:** Clinical characteristics of patients.

	All	MILD (disease score 1–2-3)	MODERATE (disease score 4)	SEVERE (disease score 5–6)
**Patients, n (%)**	67 (100)	22 (33)	20 (30)	25 (37)
**Age (years), median (IQR)**	62 (52–71)	62 (47–68)	64 (53–72)	62 (56–71)
Sex n(%)				
**Males**	44(66)	12(55)	13(65)	19(76)
**Females**	23(34)	10(45)	7(35)	6(24)
Ethnicity, n (%)				
**Caucasian**	57(85.1)	19(86)	17(85)	21(84)
**Hispanic**	9(13.4)	3(14)	2(10)	4(16)
**Afroamerican**	1(1.5)	0(0)	1(5)	0(0)
**Hospitalized patients, n (%)**	64 (95)	19 (86)	20 (100)	25 (100)
**Patients with comorbidities, n (%)**	42 (63)	11 (50)	11 (55)	20 (80)
**PaO_2_/FiO_2_ (nadir), median (IQR)**	147 (82–289)	300 (269–344)	183 (118–252)	80 (55–101)
**Temperature at admission (°C), median (IQR)**	37.5 (36.8–38.5)	37.7 (36.6–38.5)	37.1 (36.8–38.0)	37.6 (36.9–38.5)
**Hemoglobin (g/dl), median (IQR)**	13.9 (12.9–15.0)	14.0 (13.2–15.5)	14.5 (13.2–15.5)	13.3 (12.5–14.1)
**ALC (10^3^/μl), median (IQR)**	1.2 (0.8–1.4)	1.2 (1.0–1.4)	1.3 (0.9–1.6)	0.9 (0.8–1.3)
**LDH (U/l), median (IQR)**	400 (318–487)	325 (268–394)	407 (359–457)	495 (339–640)
**CPR (mg/l), median (IQR)**	110.6 (32.7–181.6)	39.1 (19.0–79.2)	119.0 (53.7–178.3)	181.6 (107.5–293.3)
**hospital admission and sampling (days post symptom onset), median (IQR)**	8 (5–12)	7 (4–12)	11 (6–12)	8 (6–14)
follow-up samples, n (%)	49 (73)	19 (86)	18 (90)	12 (48)

ALC, Absolute Lymphocyte Counts; CPR, C-Reactive Protein; FiO_2_, Fraction of inspired oxygen; IQR, Inter-Quartile Range; LDH, lactate dehydrogenase; PaO_2_, arterial Partial pressure of Oxygen.

Baseline samples were collected at the time of hospital admission with a median of 8 days (IQR 5–12) from the onset of symptoms. Follow-up samples were also analyzed for survivors at 1 (T1M), 3 (T3M) and 6 (T6M) months after hospital discharge. The results of all analysis herein described were compared to those of n=19, age- and sex-matched Healthy Donors (HD), whose blood samples were collected before the pandemic (median age 57 years; Inter-Quartile Range, IQR, 48–72 years; M/F: 11/8) by the clinicians of the Internal medicine Unit. All the healthy donors were Caucasian.

### 2.2 Altered profile of circulating monocytes associates to COVID-19 severity

**Figure 1:**
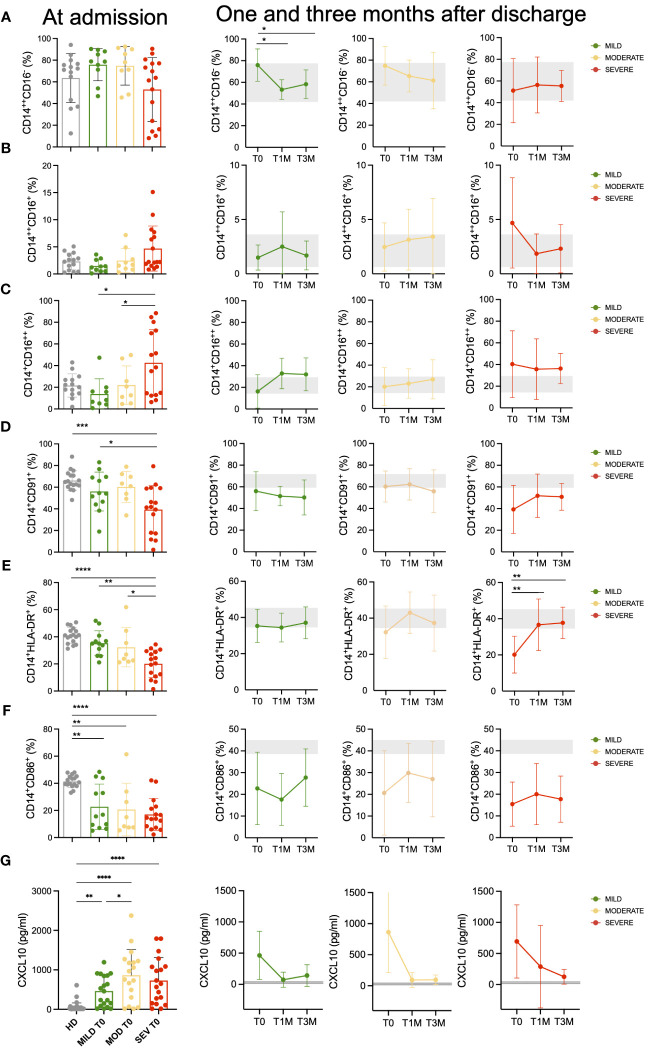
Monocytes landscape in COVID-19 patients. Monocytes phenotypic characterization of COVID-19 patients compared to age-matched healthy donors (HD). **(A-C)** Frequencies of classical (CD14^++^CD16^-^, **(A)** intermediate (CD14^++^CD16^+^, **(B)** and non-classical (CD14^+^CD16^++^, **(C)** monocytes on total CD14^+^ monocytes for Mild, Moderate (MOD) and Severe (SEV) patients at hospital admission are shown and compared to those observed in HD (left panels). Longitudinal analysis at the indicated timepoints: hospital admission (T0), 1 month (T1M) and 3 months after discharge (T3M; middle and right panels). **(D-F)** Frequencies of CD14^+^CD91^+^ **(D)**, CD14^+^HLA-DR^+^ **(E)** and CD14^+^CD86^+^ **(F)** monocytes on total CD14^+^ monocytes for Mild, Moderate and Severe patients at hospital admission are shown and compared to those observed in HD (left panels). Longitudinal analysis at the indicated timepoints: T0, T1M and T3M (middle and right panels). **(G)** Plasma levels of CXCL10 for Mild, Moderate and Severe patients at hospital admission are shown and compared to those observed in HD (left panels). Longitudinal analysis at the indicated timepoints: T0, T1M and T3M (middle and right panels). Statistics were calculated by ordinary one-way ANOVA (multiple comparisons). Asterisks represent statistical difference between each patient group and HD: * p<0.05, ** p<0.01, *** p<0.001, **** p<0.0001. Mean with SD are shown.

The proportion of circulating CD14^+^CD16^+^ cells in COVID-19 patients was within the normal range, similar to HD (data not shown). However, distinct variations in the relative composition of monocytes were observed in COVID-19 patients at admission (**Supplementary Table 2**; **Supplementary Figure 2A**). These variations suggested a trend towards a lower frequency of “classical” (CD14^bright^CD16-) monocytes in patients with severe COVID-19 (not significant; **Figure 1A**), confirmed by the accumulation of “non-classical” – also called inflammatory – (CD14^+^CD16^bright^) monocytes (35) in the same group (p<0.05; **Figure 1C**). Of notice, “non-classical” monocytes were still above the HD level at one and three months after hospital discharge in the blood of patients who experienced severe COVID-19. No significant variation was observed in the frequency of “intermediate” (CD14^bright^CD16^+^) monocytes (**Figure 1B**). Since our previous observations indicate that CXCL10 plasma concentration is associated with COVID-19 disease prognosis (30) and considering that monocyte LDL receptor-related protein (LRP1)/CD91 expressing cells are associated to the course of autoimmune-inflammatory diseases (36), we decided to analyze their potential association. Notably, monocyte LDL receptor-related protein (LRP1)/CD91 expression was significantly and selectively reduced in patients with severe COVID-19 (**Figure 1D**). Interestingly, the extent of LRP1/CD91 expression was inversely correlated with the concentration of the chemokine CXCL10 (**Supplementary Figure 2B**). At admission, MHC class II (HLA-DR) expression on monocytes was also significantly reduced in patients with severe COVID-19 compared with patients with mild or moderate disease and HD (**Figure 1E**). This change was transient and HLA-DR expression returned to the normal range three months after discharge. A significantly lower fraction of monocytes also expressed the CD86 receptor in COVID-19 patients at admission, regardless of disease severity (**Figure 1F**). The reduced CD86 expression persisted at one and three months after discharge, in agreement with previous observations (37, 38). The simultaneous reductions of HLA-DR and CD86 might impact the effectiveness of antigen presentation to CD4^+^ T cells and could reflect a dysfunctional distribution of myeloid cells (37). However, the different persistence kinetics of these changes in receptor membrane expression suggests that each reflects independent molecular events.

We determined the plasma levels of 18 cytokine and chemokines involved in COVID-19 severity (30, 31). Among these, IL-6, MIP-1b, IL-8, CXCL10, and MCP-1 (CCL2) (**Figure 1G**; **Supplementary Figure 3**) were found at higher concentrations in patients with severe or moderate disease compared to those with mild disease. These results are of special interest given the availability of biologic agents specifically targeting these pro-inflammatory molecules (39, 40). At one-month post-discharge, the levels of CXCL10 were still sustained only in patients with severe disease at hospital admission, while patients with mild or moderate acute disease had CXCL10 levels below or similar to the baseline threshold.

### 2.3 NK-cell populations are strongly affected by SARS-CoV-2 infection

**Figure 2:**
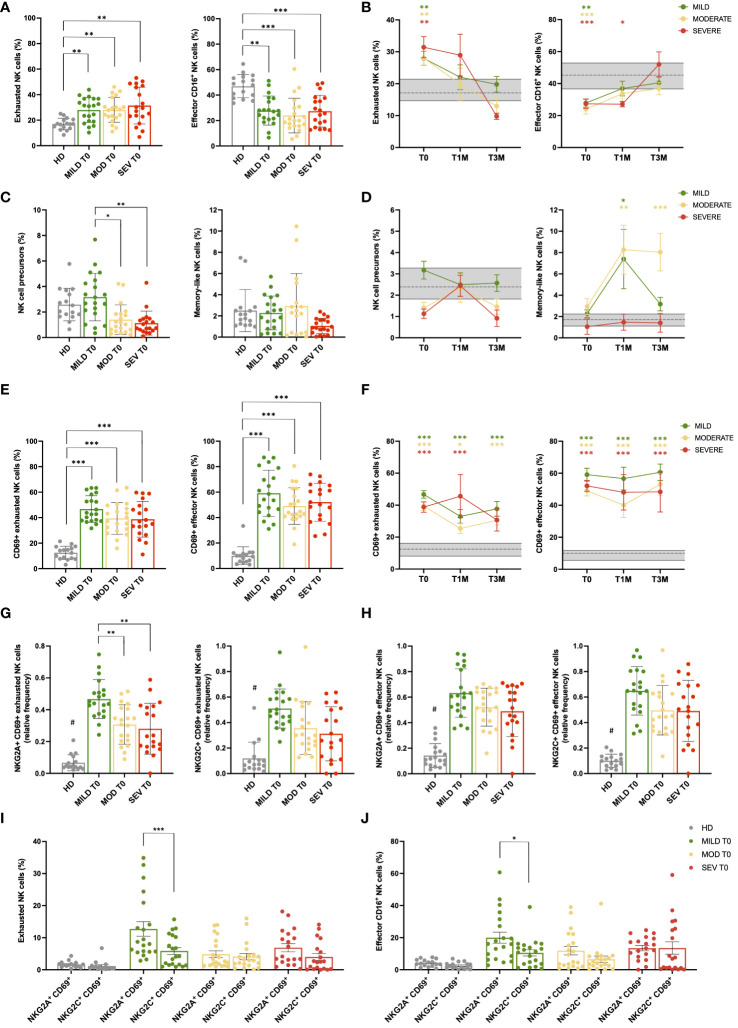
NK cell subsets are impaired during acute SARS-CoV-2 infection. **(A)** Percentage of CD56^+^ NK cell subpopulations (exhausted CD16^-^ and effector CD16^+^) in healthy donor (HD) and mild, moderate and severe COVID-19 patients, at hospital admission (T0). **(B)** Longitudinal evaluation of the percentage of exhausted and effector CD16^+^ NK cells in Mild, Moderate and Severe COVID-19 patients, at the indicated timepoints: hospital admission (T0), 1 month (T1M) and 3 months (T3M) after discharge. Follow-up samples were analyzed from 17 out of 20 mild and 16 out of 20 moderate patients enrolled in the study at T1M and/or T3M, together with 8 patients from severe group that survived to COVID-19 disease. **(C)** Percentage of NK cell precursors and memory-like NK cells in Mild, Moderate and Severe COVID-19 patients, at T0. **(D)** Longitudinal evaluation of the percentage of NK-cell precursors and memory-like NK cell subset in Mild, Moderate and Severe COVID-19 patients, at T0, T1M and T3M. **(E)** Percentage of CD69 positive cells in NK subpopulations (exhausted CD16^-^ and effector CD16^+^) of HD and Mild, Moderate and Severe COVID-19 patients, at T0. **(F)** Percentage of activated CD69^+^ cells in exhausted and effector NK subpopulations of Mild, Moderate and Severe COVID-19 patients, at T0, T1M and T3M. **(G)** Percentage of NKG2A^+^ CD69^+^ (left) and NKG2C^+^ CD69^+^ (right) exhausted NK cells in HD and Mild, Moderate and Severe COVID-19 patients, at T0. **(H)** Percentage of NKG2A^+^ CD69^+^ (left) and NKG2C^+^ CD69^+^ (right) effector CD16^+^ NK cells in HD and Mild, Moderate and Severe COVID-19 patients, at T0. Comparison between percentage of activated (CD69^+^) NKG2A and NKG2C-positive cells in both exhausted **(I)** and effector CD16^+^ **(J)** NK cells of HD and Mild, Moderate and Severe COVID-19 patients, at T0. **(A, C, E, G–J)** Error bars represent standard deviation (SD). **(B, D, F)**: Gray area represents percentage between the first and third quartiles observed in HD. The dashed gray line represents the average percentage observed in HD. Mean with SEM are shown. Statistical significance is calculated by Kurskal-Wallis’s test, following Dunn correction for multiple comparisons. Asterisks represent statistical difference between each patient group and HD. * p<0.05; ** p<0.01; *** p<0.001; # p < 0.001 of HD population vs all COVID-19 patients. **(I, J)** Asterisks highlight significant differences by Mann-Whitney’s test.

Patients with COVID-19 were investigated for the main NK-cell subpopulations as identified according to the differential expression of CD16 and CD56 (**Supplementary Table 3**; **Supplementary Figure 4**). While CD56 is a specific marker of NK cells, levels of CD16 reflect their cytotoxic capacity, low expressions being associated to reduced residual cytotoxic capabilities due to functional exhaustion (41, 42). At T0, we observed that the frequencies of the different NK-cell subsets were significantly affected. Specifically, COVID-19 patients exhibited a significant increase (p<0.01) in the percentage of NK cells displaying an exhausted, anergic phenotype (CD16^-^CD56^dim^) and, consequently, were characterized by a dramatic decrease (p<0.01) in the percentage of the classical CD16^+^CD56^dim^ effector population compared to HD (**Figure 2A**). Conversely, the CD16^dim^CD56^dim^ effector subset, which represents an unclassical subpopulation with distinctive cytotoxicity characteristics compared to the CD16^+^CD56^dim^ counterpart (41), showed a significant increase in patients with severe disease compared to those with moderate disease (p<0.01, **Supplementary Figure 5A**). In follow-up samples, all COVID-19 patients showed a progressive and significant reduction of the exhausted NK-cell component as they reached levels similar to HD at T3M (**Figure 2B**). Interestingly, in mild and moderate patients the normal frequency of classical CD16^+^ effector NK cells was restored at T1M, whereas in severe patients such cell population recovered its physiological range only at T3M, suggesting a more profound and persistent impoverishment of the effector NK-cell component (**Figure 2B**). The CD16^dim^CD56^dim^ effector subset did not undergo significant changes over time after disease resolution (**Supplementary Figure 5B**), although its number was slightly higher in patients with severe disease compared to the other severity groups and HD (**Supplementary Figure 5B**). Strikingly, we found that CD16^-^CD56^bright^ NK cells, commonly associated with a precursor phenotype, were significantly reduced at T0 in patients with moderate (p<0.05) and severe respiratory distress (p<0.01), as compared with patients with mild pneumonia. Conversely, no difference was observed for memory-like (CD56^-^/CD16^bright^) NK cells at T0 (**Figure 2C**), whereas they increased in both mild and moderate patients after disease resolution (**Figure 2D**). Finally, during follow-up, the percentage of NK-cell precursors did not display significant differences amongst the three groups of patients and compared with HD (**Figure 2D**).

### 2.4 NK cells remain activated for up to 3 months after SARS-CoV-2 infection, while mild patients displayed highest NKG2A^+^ cells during acute disease

To characterize NK cell activity, the markers CD69, NKG2A, and NKG2C were employed. Specifically, NKG2A and NKG2C are receptors that act as pivotal inhibitory and activator immune checkpoints, respectively, while CD69 is a well-established marker of activation and tissue migration of NK cells (43–45). Given the relevance of NKG2A and CD69 in NK cell maturation, function, and metabolism (46), the co-expression of NKG2A and CD69 was investigated to determine the impact of NK cells with migratory capacity (CD69+) on infected tissue, since NKG2A, by acting as a checkpoint inhibitor, is protective for target cells by limiting the NK cell-mediated cytotoxic response and mitigating tissue damage (47). A sustained and significant increase of NK cells expressing the activation and tissue migration marker CD69 occurred in COVID-19 patients regardless disease severity in both the exhausted and effector NK-cell subpopulations compared to HD (p<0.001; **Figure 2E**; **Supplementary Figure 5C**). Despite the exhausted/effector ratio returned to its physiological value after disease resolution, CD69^+^ NK cells persisted during the entire follow-up period, suggesting that NK-cell inflammatory activity was maintained for at least 3 months after SARS-CoV-2 acute infection (**Figure 2F**; **Supplementary Figure 5D**). Interestingly, the percentage of NKG2A^+^ CD69^+^ and NKG2C^+^ CD69^+^ NK cells was also significantly increased in both exhausted and CD16^+^ effector NK-cell subpopulations when compared to HD (p<0.001 COVID-19 patients vs HD; **Figures 2G-H**). Noteworthy, patients with mild disease displayed the highest percentage of activated NKG2A^+^ cells within the exhausted NK subpopulation, thus resulting in a significant increase compared to both moderate and severe groups (p<0.01 mild vs moderate/severe, **Figure 2G**). Moreover, in patients with mild disease, NKG2A^+^ CD69^+^ cells significantly outnumbered the NKG2C^+^ CD69^+^ counterpart, both within exhausted (p<0.001) and CD16^+^ effector NK cells (p<0.05, **Figures 2I, J**), highlighting an important shift towards NKG2A-mediated inhibitory signals in tissue-migrating cells. After 3 months, activated NKG2A^+^ and NKG2C^+^ cells were still significantly higher in all COVID-19 patients compared to HD, although the differences between NKG2A^+^ CD69^+^ and NKG2C^+^ CD69^+^ subsets were no longer significant in the mild cohort at T3M, as well as in the other disease groups (data not shown).

### 2.5 Severe COVID-19 causes a progression of B cells toward a terminal differentiation stage

A comprehensive high dimensional flow analysis of B cells was performed to study the phenotypic changes of circulating B cells upon COVID-19. We designed a 21 color-based panel (**Supplementary Table 4**) that longitudinally describes the differentiation stage, functional status, and migratory potential of B cells in peripheral blood in COVID-19 patients with different degrees of severity.

**Figure 3:**
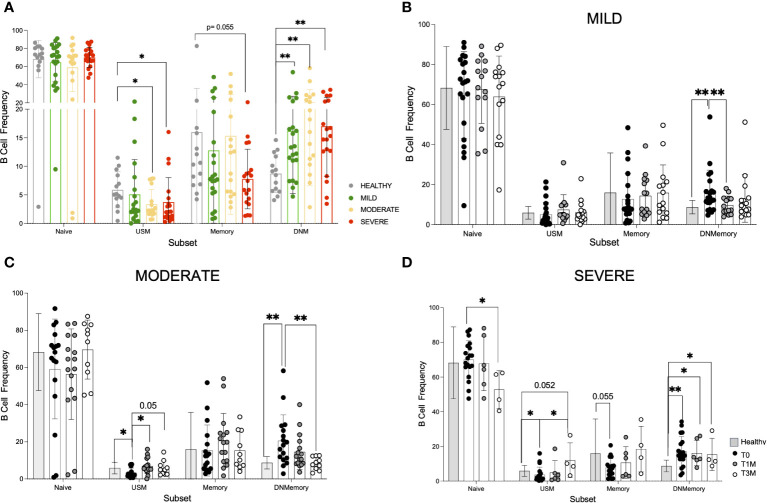
Phenotype dynamic of peripheral B cells in COVID-19 patients. A 21-color based panel was designed to define B cell phenotype by flow cytometry from 14 healthy donors and COVID-19 patients at T0, T1M, and T3M from hospital discharge with mild (21 pts at T0, 14 pts at T1M, 15 pts at T3M), moderate (17 pts at T0, 16 pts at T1M, 10 pts at T3M) or severe infection (19 pts at T0, 6 pts at T1M, 4 pts at T3M). B cell subsets were defined as naïve CD27^-^IgD^+^, unswitched memory (USM) CD27^+^IgD^+^, memory CD27^+^IgD^-^ and double negative memory (DNM) CD27^-^IgD^-^. **(A)** B cell frequency in Naive, USM, Memory and DNM B cell subsets from healthy donors, mild, moderate and severe patients at hospital admission. B cell frequency in the same subsets as in A at T0, T1M, and T3M is reported for **(B)** mild **(C)** moderate **(D)** severe patients (dots as indicated) in comparison with healthy donors (grey bar). Statistics were calculated by tTest multiple comparison: * p<0.05, ** p<0.01. Standard Deviation is reported for each data set.

The study examined B-cell differentiation stages, including CD27^-^IgD^+^ Naïve, CD27^+^IgD^+^ unswitched memory (USM containing both IgM^+^IgD^+^ or more rarely IgD^+^IgM^-^ subsets), and CD27^+^IgD^-^ memory among which IgG^+^ are defined as switched memory and CD27^-^IgD^-^ as double negative memory (DNM; **Supplementary Figure 6A**). Regulatory B cells (Bregs) with immune suppressive function are contained in the CD38^++^ CD24^++^ population, while CD38^++^CD24^-^ plasmablasts are reported to be precursors of CD38^++^CD24^-^CD138^+^ antibody-secreting plasmacells (48) (**Supplementary Figure 6A**). At T0, COVID-19 led to a significant increase of CD27^-^IgD^-^ DNM cells and decrease of CD27^+^IgD^+^ USM B-cell frequency in patients with moderate and severe disease, while classical memory B-cell percentage tended to decrease only in patients affected by severe disease (**Figure 3A**). Interestingly, CD27^+^IgD^+^ USM and classical memory B-cell frequency returned to normal levels within T3M in all the patient cohorts, while only in presence of severe disease CD27^-^IgD^-^ DNM cells persisted with higher frequency and the naïve B-cell subset was reduced if compared to HD (**Figures 3B-D**). By contrast, the frequency of CD38^++^CD24^++^ Breg cells and of CD38^++^CD24^-^ plasmablasts appeared to be unaffected by infection (not shown). In-depth phenotype analysis of the DNM subset in patients at T0 (**Supplementary Figure 6B**) revealed a lower frequency of IgG^+^ B cells and a more pronounced IgM^+^ population than in HD. The activation marker CD69 was expressed at higher level in all infected individuals, while patients with severe disease were characterized by the lack of CD21 BCR co-receptor, indicating B-cell exhaustion upon chronic activation (49), and of CXCR5 chemokine receptor, pointing to a defective entry in B-cell follicles (50). Moreover, lower expression of CD80 and CD40 co-stimulatory molecules was observed in patients along with low or undetectable CD1c molecule expression presenting lipid antigens to unconventional T-cell subsets (51). Collectively, these data suggested that severe COVID-19 causes a progression of B cells towards an activated/exhausted DNM differentiation stage with impaired homing potential to lymph node and reduced capability to functionally interact with T cells. While these defects persisted for 3 months after discharge in severe patients, they did not emerge nor significantly declined in mild and moderate disease.

### 2.6 Patient-specific T-cell metaclusters with differential memory, exhaustion and activation phenotypes are associated with severe COVID-19

T cells were quantified and evaluated for the expression of lineage, activation, differentiation, and exhaustion markers (**Supplementary Table 5**). At onset, severely ill patients were lymphopenic (ALC<10^3^/μl, **Table 1**), with reduced frequencies of total T cells (p<0.05) and similar proportions of both CD4 and CD8 subsets on T cells (**Supplementary Figures 7A-C**). All COVID-19 patients displayed a lower proportion of T regulatory cells (CD4^+^CD25^bright^CD127^low^; Tregs) compared to HD, independently of the disease category (p<0.0001, **Supplementary Figures 7D, E**), and such difference decreased over time (**Supplementary Figures 7F, G**).

**Figure 4:**
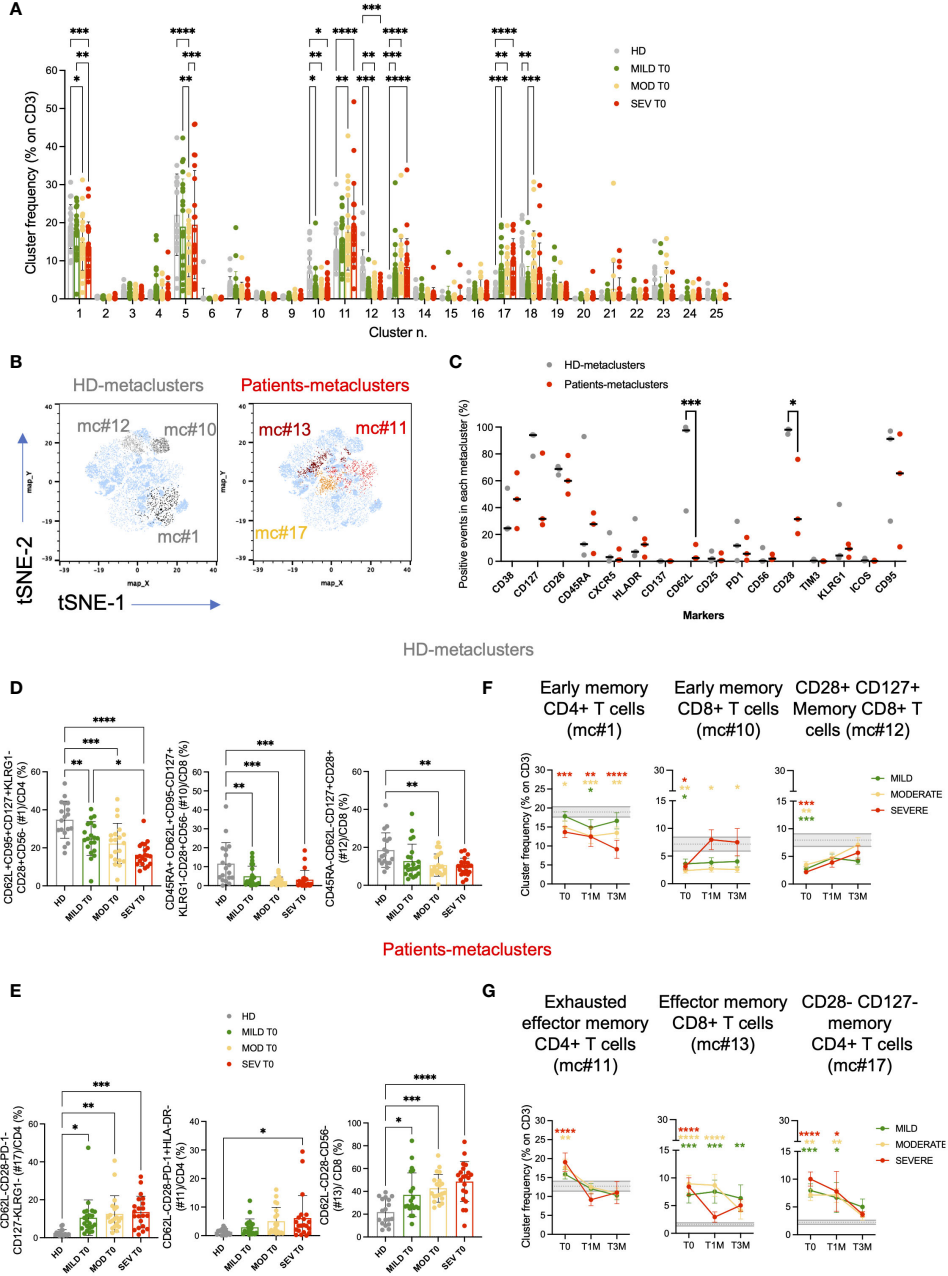
High dimensional analysis of FACS data revealed the presence of several T-cell clusters deregulated in COVID-19 patients. T-cell phenotypic characterization of COVID-19 patients compared to age-matched healthy donors (HD; unsupervised high dimensional analysis). **(A)** metaclusters frequencies on total CD3+ cells for Mild, Moderate and Severe patients at hospital admission (T0) are shown and compared to those observed in HD. **(B)** t-SNE maps and the overlay of HD-specific (left; light and dark gray) and patients-specific (right; yellow and red) metaclusters. **(C)** percentage of events positive for the indicated markers in HD- and patients-specific metaclusters. Unsupervised high dimensional analysis and clusterization of CD3+ events were performed by cytoChain. **(D, E)** Validation of the phenotypic signatures associated to HD **(D)** and patients **(E)** -specific metaclusters by manual gating. Metacluster frequencies are shown for Mild, Moderate and Severe patients and compared to HD at hospital admission (T0). Mean with Standard deviation are shown. Statistical analysis in **A** and **C** were performed by 2way ANOVA (multiple comparisons). Statistical analysis in **D** and **E** were performed by ordinary one-way ANOVA (multiple comparisons). **(F, G)** Longitudinal analysis of the frequencies of the metaclusters of interest on CD3^+^ T cells at the indicated timepoints: hospital admission (T0), 1 month (T1M) and 3 months (T3M) after discharge for HD **(F)** and COVID-19 **(G)**-specific metaclusters. Frequencies are shown for Mild (green), Moderate (yellow) and Severe (red) patients and compared to HD. Grey area represents frequencies of each metacluster on CD3^+^ T cells between the first and third quartiles observed in HD. The dashed gray line represents the average percentage observed in HD. Mean with SEM are shown. Statistical analysis were performed by 2way ANOVA (multiple comparisons). Asterisks represent statistical difference between each patient group and HD. * p<0.05; ** p<0.01; *** p<0.001; **** p<0.0001.

High dimensional analysis and clustering of T cells by cytoChain (52), a novel dataset mining tool, were performed on mild, moderate and severe patients at different timepoints and on HD for a total of 154 samples. A unique t-SNE map was generated and revealed the presence of 3 HD- and 3 patients-specific metaclusters (**Figures 4A, B**) that were further characterized. Interestingly, when evaluating the expression profile of each T-cell marker, we found that patients-specific metaclusters showed a lower proportion of CD62L^+^ (p<0.001) and of CD28^+^ (p<0.05) cells compared to HD-specific metaclusters (**Figure 4C**), indicating a more differentiated phenotype associated to the disease. Both markers drove the metacluster segregation, as shown by the PCA analysis and marker maps (**Supplementary Figures 8A, B**). Patients-specific metaclusters displayed a trend for higher expression of CD38 (median 46.28% vs 24.64% in HD-metaclusters) and HLA-DR (median 12.51% vs 7.12% in HD-metaclusters), suggesting that these T cells were recently activated. CD127, the IL-7 receptor α chain, and CD26, an extracellular peptidase expressed at high levels by memory T cells (53) and able to orchestrate the migratory response of T cells during inflammation (54) appear more expressed in HD-specific than in patients-specific metaclusters, confirming the highly differentiated effector phenotype of the latter. The exhaustion marker TIM-3, and the costimulatory molecules CD137 and ICOS were barely detectable in HD- or patient-specific metaclusters.

Each of the 6 differentially expressed metaclusters had a specific phenotypic signature, as highlighted by the heatmap (**Supplementary Figure 8C**) and relevant signatures were validated by manual gating (**Supplementary Figure 9**). Metacluster #1 was more frequent in healthy donors than in COVID-19 patients and was characterized by CD4+ T cells expressing early differentiation markers (CD62L^+^CD95^+^CD127^+^CD28^+^) and low levels of KLRG1 and CD56 (**Figure 4D**; **Supplementary Figure 9A**). In metacluster #1 we retrieved also CXCR5^+^ CD4^+^ T cells (21.26% on total events), a marker typical of circulating follicular helper T cells (cTfh, **Supplementary Figure 9B**). Indeed, the frequency of cTfh was lower in COVID-19 patients than in healthy donors (**Supplementary Figure 8D**). Notably, the second HD-specific metacluster (#10, CD45RA^+^CD62L^+^CD95^-^CD127^+^KLRG1^-^CD28^+^CD56^-^) was enriched in Naive CD8^+^ T cells (**Figure 4D**; **Supplementary Figure 9C**), while #12 included CD45RA^-^CD62L^-^ T cells expressing high levels of CD28 and CD127, suggestive of a short replicative history and maintenance of memory and effector functions (55) (**Figure 4D**; **Supplementary Figure 9D**). On the contrary, metacluster #17 showed a phenotype typical of effector/effector memory CD4^+^ T cells (CD62L^-^CD28^-^PD-1^-^CD127^-^KLRG1^-^) and was more frequent in all disease categories than in HD (**Figure 4E**; **Supplementary Figure 9E**). Moreover, metacluster #11 was characterized by the exhausted CD4^+^ T cells (CD62L^-^CD28^-^PD-1^+^HLADR^-^) signature, which was significantly more expanded in Severe patients than in HD (p<0.05; **Figure 4E**; **Supplementary Figure 9E**). Similarly, in the CD8 compartment, patients-specific metacluster #13 with a phenotypic signature of T_EM_/T_EMRA_ (CD62L^-^CD28^-^CD127^-^CD56^-^) was enriched across all disease categories (**Figure 4E**; **Supplementary Figure 9F**). After manual gating, we observed a high expression of both CD38 (46.27% on total events) and of HLA-DR (16.72% on total events) in metacluster #13. Indeed, the frequency of late differentiated and activated T cells co-expressing CD38 and HLA-DR was particularly expanded in patients with severe disease compared to HD (p<0.01, **Supplementary Figures 8E**, **9F**).

Altogether, results of the unsupervised analysis suggested that COVID-19 correlated with a low expression of CD62L, CD28 and CD127, a hallmark of highly differentiated and short living effector cells (T_EM_ or T_EMRA_). Notably, severe disease was associated with the increased expression of the exhaustion marker PD-1 on CD4^+^ and of activation markers on CD8^+^ T cells.

Analysis of PBMC at 1 and 3 months after hospital discharge confirmed a persistent T-cell perturbation (**Figures 4F, G**). At T3M, among the 6 differentially expressed metaclusters, #1 (including early differentiated CD4^+^ T cells and cTfh cells) and #10 (including early differentiated CD8^+^ T cells) were still reduced in Moderate/Severe but not in Mild patients, compared to HD. Conversely, more differentiated effector memory CD8^+^ T cells (metacluster #13) remained enriched also in patients with mild symptoms. Together, these data supported a prolonged alteration of T-cell homeostasis in patients affected by COVID-19.

### 2.7 High levels of neutralizing S1-specific IgA are associated with severe COVID-19 clinical course

**Figure 5:**
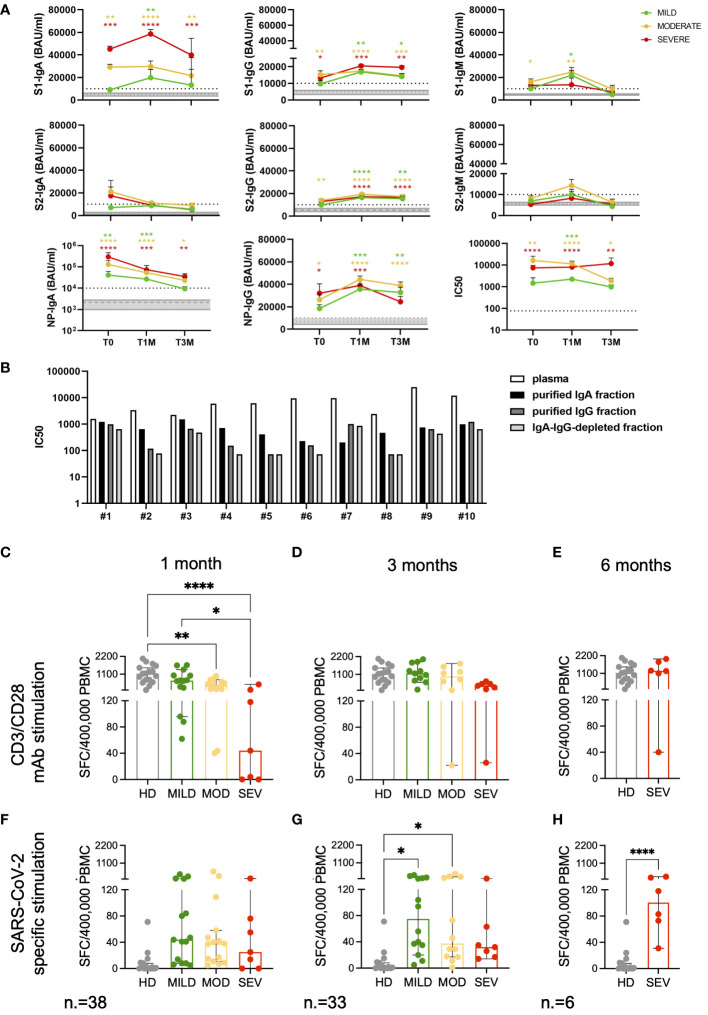
Magnitude and kinetics of SARS-CoV-2 specific humoral and T-cell responses. Longitudinal analysis of SARS-CoV-2 specific antibody levels (BAU/ml) and neutralizing antibody titers (IC50) **(A)** in mild, moderate and severe COVID-19 patients at T0, T1M and T3M. Mean with SEM are shown. Gray area represents the values measured in HD included between the first and third quartiles. The dashed gray line represents the average value observed in HD. The dashed black line represents the cutoff of the ELISA tests (10000 BAU/ml) or the lowest dilution (1:77.7) of the neutralization assays. Statistical analysis was performed by Kruskal-Wallis test, following Dunn correction for multiple comparisons. Asterisks represent statistical difference between each patient group and HD. * p<0.05; ** p<0.01; *** p<0.001; **** p<0.0001. **(B)** IC50 of IgA- and IgG-purified fractions and IgA-IgG-depleted fractions from plasma collected from ten randomly selected moderate and severe patients. Functional T-cell responses measured by IFN-γ ELISpot at the indicated timepoints after stimulation with CD3 and CD28 specific antibodies **(C-E)** and SARS-CoV-2 Spike and Nucleocapsid peptide pools **(F-H)**. Results are expressed as spot forming cells (SFC)/400’000 PBMC. Frequencies are shown for Mild (green), Moderate (yellow) and Severe (red) patients and compared to HD (grey). Median with 95% CI are shown. Statistical analysis in **(C, D, F, G)** were performed by ordinary one-way ANOVA (multiple comparisons). Statistics in **(E, H)** were performed by unpaired tTest. * p<0.05; ** p<0.01; **** p<0.0001.

The magnitude of the SARS-CoV-2 specific antibody response was examined in a cohort of 39 COVID-19 patients from our study, composed of 15 with mild symptoms, 14 with moderate symptoms and 10 with severe symptoms who survived to the disease. We measured levels of IgM, IgG and IgA directed against SARS-CoV-2 S1, S2 and NP antigens (**Figure 5A**).

At T0, the antibody response was heterogeneous within the cohort, irrespective of disease severity. IgM antibodies, indicative of early response to viral infection preceding the class-switched IgG response, responsible for the long-term immunity and immunological memory, were detected. The IgA response, which generally exceeds levels of IgM in serum and is significantly more present in mucosal surfaces and secretions (56), was also evaluated. We found that SARS-CoV-2 infection triggered an unconventional antibody response.

In this cohort, only patients with moderate symptoms developed elevated levels of S1-specific IgM compared to HD (p=0.0253) at the onset of the disease. Both SARS-CoV-2 reactive IgA and IgG were detected at T0, concomitantly with IgM, in both moderate and severe patients. Of note, mild patients did not develop statistically significant levels of SARS-CoV-2 specific antibodies compared to HD, except for NP-IgA (p=0.0053) at the onset of the disease. A significant increase in NP-IgA levels was observed in moderate and severe patients as well (p<0.0001), together with IgA towards S1 (p=0.0042 and p=0.0004, respectively), suggesting a strong association between IgA development and disease severity.

The magnitude of the IgG response was lower than that of the IgA response in all COVID-19 patients. IgG targeting NP and S proteins (both S1 and S2 subunits) were higher in moderate patients at T0 than in HD (p=0.0273 for NP-IgG; p=0.0097 and p=0.0028 for S1- and S2-IgG, respectively), whereas severe patients developed S1- (p=0.0341) and NP-IgG (p=0.0118). Overall, S1 and NP appeared to be the most immunogenic antigens triggering specific antibody responses at onset in moderate-severe COVID-19.

A delayed S1-specific IgM response was detected in mild patients at T1M (p=0.0161). S1-IgM increased in moderate patients (p=0.0014), whereas their levels were undetectable at T3M in all patients’ categories.

The robust NP-IgA response observed at T0 in all patients was followed by a significant drop at later time points, although their levels remained higher compared to HD (p=0.0004, p<0.0001, p=0.0001 for mild, moderate and severe patients, respectively, at T1M; p=0.6420, p=0.0118, p=0.0088 for mild, moderate and severe patients, respectively, at T3M).

All patient groups increased their S1-IgA levels at T1M compared to HD (mild: p=0.0015, moderate: p<0.0001, severe: p=0.0003) and, although there was a decrease, these levels remained high in moderate (p=0.0003) and severe (p=0.0019) patients only at T3M.

The kinetics of S1- and S2-IgG showed a similar increase at T1M, with only a slight decrease at T3M, confirming that SARS-CoV-2 S-IgG provide long-term protection in COVID-19 patients, irrespective of disease severity. A similar trend was observed for IgG targeting NP.

We detected S-specific neutralizing activity at T0 only in plasma from patients with moderate and severe symptoms. This is in line with the absence of significant levels of antibodies targeting the S protein in mild patients. We found that, together with IgG, IgA contributed to the early neutralizing response in moderate and severe patients (**Figure 5B**). Indeed, 8 out of 10 IgA-purified fractions showed higher neutralizing titers compared to the IgG-purified counterparts. The presence of soluble factors produced during the cytokine storm triggered in COVID-19 patients, as well as IgM, needs also to be taken into account as the IgA-IgG-depleted fractions showed neutralizing activity as well. At later time points, the neutralizing titers increased in all patients, with a slight decrease at T3M, mirroring the levels of binding antibodies.

### 2.8 Strength and kinetics of SARS-CoV-2 specific T-cell responses inversely associate to disease severity

Both polyclonal and SARS-CoV-2 specific T-cell responses were evaluated in 40 patients and in 17 age-matched HD by means of IFN-γ ELISpot. At one, but not at three months after discharge, polyclonal T-cell responses in patients affected by moderate to severe forms of the disease were lower than those observed in HD (p<0.01 and p<0.0001, respectively) and in mild patients (p<0.05, **Figures 5C-E**). At 3 months, SARS-CoV-2 specific T-cell frequencies were enriched in mild/moderate (p<0.05) but not in severe patients, compared to unexposed HD (**Figures 5F, G**). IFN-γ production against SARS-CoV-2 viral proteins was observed in severely ill patients only when an additional follow-up timepoint was evaluated at 6 months after discharge (p<0.0001, **Figure 5H**). These results indicate a link between impaired T-cell response to SARS-CoV-2-viral antigens in peripheral blood and severe COVID-19 disease.

### 2.9 NK-cell precursors, CD14^+^CD91^+^ monocytes and CD8^+^ effector memory T cells are the most robust immunological correlates of COVID-19 clinical course

**Figure 6:**
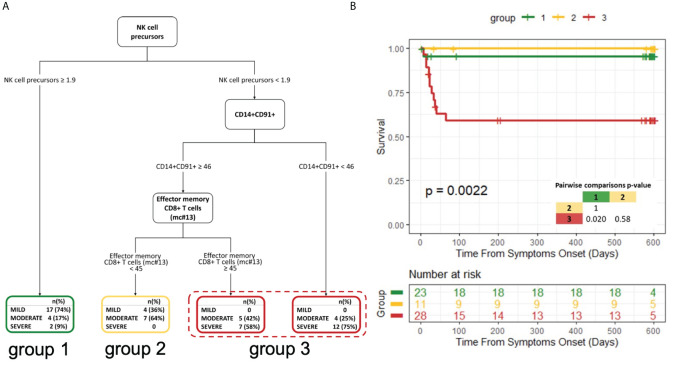
Decision tree for the classification of disease severity and its impact on patient survival **(A)** Decision tree for the classification of disease severity. **(B)** Kaplan−Meier curves and log-rank test for comparing the three groups obtained from the decision tree analysis. The three groups were defined based on the highest frequently category in the final nodes of the decision tree. P-value of pairwise comparisons were adjusted with Bonferroni’s correction.

We next wondered which immunological variables had the strongest impact on COVID-19 severity and outcome. Taking advantage of the availability of several clinical, laboratory and immunological parameters collected simultaneously at hospital admission, we estimated a decision tree for the classification of disease severity. Only variables showing a significant association with severity groups in univariate analysis were considered (**Supplementary Table 6**). Out of more than 60 input variables, NK-cell precursors, CD14^+^CD91^+^ monocytes and CD8^+^ Effector Memory T cells (mc#13) were identified by the classification tree model as those best discriminating between disease severity groups (**Figure 6A**). Having NK-cell precursors frequency >1.9% allowed the classification of 74% of patients with mild symptoms. On the contrary, most patients (75%) with <1.9% NK-cell precursor and <46% CD14^+^CD91^+^ monocyte frequencies experienced the worst outcomes characterized by severe symptoms. Among patients with low frequencies of NK-cell precursors but high frequencies of CD14^+^CD91^+^ monocytes, the level of CD8^+^ Effector Memory T cells (mc#13) could discriminate between different disease severity. Patients with <45% CD8^+^ Effector Memory T-cell frequency experienced mild (36%) and moderate (64%) symptoms, while patients with >45% CD8^+^ Effector Memory T-cell frequency experienced moderate (42%) and severe (58%) symptoms.

We further explored the correlations of the variables selected by the decision tree with all the input variables at T0 (**Supplementary Figure 10**). The frequency of NK-cell precursors was inversely correlated with the frequency of effector CD16^+^ NK cells and with the neutralizing activity of specific antibodies. CD14^+^CD91^+^ monocytes were directly correlated with circulating immunostimulatory monocytes (CD14^+^HLA-DR^+^) and the frequency of classical monocytes, but an inverse correlation with the level of RBD-specific IgM and the frequency of Non-classical monocytes was observed. Besides being correlated with other T-cell subsets, CD8^+^ Effector Memory T cells (mc#13) inversely correlated to the frequency of USM B cells, which have been recently described as a biomarker of a shorter duration of COVID-19 (57), and notably with the nadir oxygenation index (P/F).

Next, based on the highest frequent category in the final nodes of the decision tree, we grouped leaves to create three distinct groups. Survival analysis showed a marked difference among the three groups (p=0.0022; **Figure 6B**).

In summary, patients with a lower frequency of NK-cell precursors and CD14^+^CD91^+^ monocytes and a higher frequency of CD8^+^ Effector Memory T cells had a lower probability to survive.

### 2.10 Respiratory disease at hospital admission and a lower frequency of Bregs during follow-up are associated with the occurrence of long COVID at 6 months

The same set of clinical, laboratory, and immunological variables collected at hospital admission (**Supplementary Table 7**) was included in a classification tree analysis aimed at discriminating patients based on their risk of experiencing long COVID at 6 months. Long COVID was defined as the persistence of at least one physical symptom after 12 weeks from acute disease, not explained by any other cause. These symptoms included dyspnea, smell or taste alterations, brain fog, myalgias, arthralgias, fatigue, chest pain, cough, headache, insomnia, anxiety, and post-traumatic stress disorder, as assessed during the 6-month follow-up visit (**Supplementary Table 8**). In our cohort, 22 (32.8%) developed long COVID at six months after hospital discharge.

**Figure 7:**
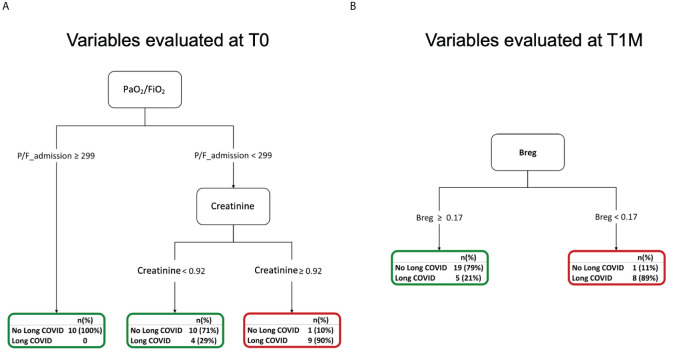
Decision trees for the occurrence of long COVID at 6 months **(A)** Decision tree for the occurrence of long COVID at 6 months considering variables evaluated at T0. **(B)** Decision tree for the occurrence of long COVID at 6 months considering variables evaluated at T1M.

Out of more than 60 input variables, lower values of the oxygenation index (PaO_2_/FiO_2_) and higher values of creatinine at admission were associated to a higher risk of experiencing long COVID at 6 months (**Figure 7A**). Specifically, a PaO_2_/FiO_2_>299, above the cut-off value for diagnosing a respiratory disease (**Supplementary Figure 1A**), allowed classifying 100% of patients who did not develop long COVID at 6 months. On the contrary, most of the patients (90%) showing <299 PaO_2_/FiO_2_ (mild/severe respiratory disease) and >0.92 mg/dl Creatinine at T0 experienced the persistence of physical symptoms long-term. Increasing values of creatinine indicate kidney damage during the acute phase of the disease.

Immunological variables were longitudinally evaluated at 1 month after hospital discharge. We considered values collected at T1M in a classification tree analysis to discriminate patients who experienced long COVID at 6 months (**Supplementary Table 9**). Among more than 50 input variables, the altered frequencies of Bregs observed at T1M was associated with the development of long COVID. Indeed, at this timepoint, having Breg frequency <0.17% allowed predicting 89% of patients with long COVID (**Figure 7B**). The lack of Breg cells and of their well-established immune regulatory function might contribute to the reported persistence of systemic inflammation and higher incidence of auto-immune diseases associated with long COVID (58, 59).

## 3 Discussion

In this study we systematically examined various cell subsets and markers of both innate and adaptive immunity as putative factors associated with COVID-19 severity. First, univariate analysis allowed us to pre-select variables showing a significant association with different disease severity groups. Thanks to the availability of samples collected longitudinally over time, their kinetics were also described during a follow-up period of 3 months after hospital discharge. Next, we implemented a decision tree that enabled us to perform a multiparametric analysis of the clinical course of COVID-19, considering the main clinical, laboratory, and biological variables collected simultaneously upon admission. Out of a pool of more than 60 parameters, we identified NK-cell precursors, CD14^+^CD91^+^ monocytes, and CD8^+^ Effector Memory T cells as those variables with the highest impact on COVID-19 severity and patient survival. Finally, enrolled patients were monitored for the occurrence of long COVID at 6 months after discharge, according to international definitions (https://www.cdc.gov/coronavirus/2019-ncov/long-term-effects). Main clinical and biological variables evaluated at the different timepoints were challenged for the association with the persistence of physical symptoms in COVID-19 survivors (60, 61).

The monocyte landscape was described in relation to COVID-19 severity. Non-classical monocytes were expanded in patients affected by severe COVID-19 compared to milder categories. This expansion suggests an increased transition from classical to intermediate to non-classical monocyte, a phenomenon previously observed in a model of systemic inflammation (62). The unbalanced distribution of the main monocyte subsets upon inflammatory conditions might be explained with the migration of classical monocytes towards inflamed tissues (63, 64) – these cells are in fact considered as composing the “macrophage precursor pool” (65) – and by the loss of CD16 expression after stimulation by infectious or pro-inflammatory agents (35) as it occurs in COVID-19. On the other hand, the frequency of HLA-DR^+^, CD86^+^ and CD91^+^ monocytes was dramatically reduced during COVID-19 and this contraction was directly proportional to the severity of the disease. While the low expression of HLA-DR (hallmark of monocytic-MDSC) and CD86 on monocytes has previously been associated with a state of general immune paralysis in critically ill patients (12) and to a lower ability to control bacterial superinfections (66), the CD14^+^CD91^+^ subset was less investigated in COVID-19. Interestingly, LRP1/CD91 gene deletion in myeloid cells has been shown to exacerbate the response to LPS through the release of pro-inflammatory cytokines (67). A reduced frequency of CD14^+^CD91^+^ cells has also been associated with inflammatory conditions (68). Furthermore, the ApoE-CD91 interaction on myeloid cells has been linked to the resolution of lung fibrosis in mice (69). In our series, the proportion of LRP1/CD91 expressing monocytes was reduced in severe cases and was inversely correlated with the level of CXCL10, selected as a representative cytokine in the storm observed in COVID-19 patient sera. Thus, the hyperinflammatory environment observed in critically ill patients was associated to a profound myeloid dysfunction as reflected by an impaired monocyte phenotype.

Next, the distribution and activation status of NK-cell subpopulation was investigated over time. A dysregulated NK-cell response is a hallmark of severe COVID-19 in which NK cells showed a reduced cytotoxicity and an accumulation of CD56^dim^ terminally differentiated NK cells (70, 71). Accordingly, we found a significant increase of NK cells that exhibit an exhausted phenotype (CD16^-^CD56^dim^) in all COVID-19 patients compared to age-matched healthy controls, and of intermediate CD16^dim^CD56^dim^ effector population particularly in the severe group. In contrast, CD16^-^CD56^bright^ NK-cell precursors were significantly reduced in patients showing moderate to severe symptoms, as compared with patients experiencing mild pneumonia. This subset produces large amounts of cytokines, including IFN-γ, and is considered critical for the development of an effective adaptive response against viruses during the early phase of the immune response (72, 73). Strikingly, the frequency of CD16^-^CD56^bright^ NK-cell precursors emerged as one of the strongest variables able for distinguishing different clinical outcomes at hospital admission as demonstrated by the decision tree in **Figure 6**. In line with the hypothesis that NK-cell compartment may possess immunomodulatory abilities, patients with mild symptoms presented the highest fraction of inhibitory NKG2A^+^CD69^+^ cells within the exhausted NK subpopulation. Moreover, all COVID-19 patients showed a higher proportion of activated CD69^+^ exhausted and effector NK subpopulations compared to healthy controls, suggesting elevated cytotoxic activity of CD56^dim^ NK cells. Interestingly, the NK-cell activation status persisted beyond the acute phase of COVID-19 for up to 3 months after hospital discharge. Finally, we observed an increase of the memory-like CD16^+^/CD56^-^ NK-cell compartment after resolution of the mild and moderate forms of disease. However, further evaluations are needed to confirm the presence of clones specific for SARS-Cov-2 within this NK-cell subset and to support evidence of an adaptive response and increased protection from re-infection, as recently suggested (74, 75).

We then explored the two branches of the adaptive immune response, B and T lymphocytes. In severe cases of COVID-19, B cells were characterized by an activated/exhausted terminal differentiation stage with impaired homing potential to lymph nodes and capability to functionally interact with T cells. Unlike patients with milder symptoms, B cells maintained this dysfunctional phenotype for up to 3 months after discharge in severe cases, indicating the development of an impaired memory response. This may be related to inefficient T-B cell interaction within the germinal center, as early memory CD4 T cells were reduced in all patients with a more pronounced reduction in severe cases. Accordingly, circulating T follicular helper (cTfh) cells were also reduced in all patients. Collectively, these findings are quite consistent with published evidence showing significant reduction of circulating class-switched (IgD^−^CD27^+^), not-class-switched (IgD^+^CD27^+^) memory B cells and Bregs, and a robust increase of CD27^−^IgD^−^ B cells and, in about 60% of patients, also of CD27^+^CD38^+^ plasmablasts (76, 77).

The effectiveness of antibodies-mediated immunity relies on their affinity, quantity and class or isotype that are able to trigger other immune effector functions, including the activation of the complement cascade or of immune cells expressing the proper Fc receptors (78). The features of the SARS-CoV-2-specific antibody response appear heterogenous among individuals. It is well established that the virus triggers an unconventional response (79), in which the concomitant development of the three immunoglobulin subtypes early during the infection represents a hallmark. In our cohort, patients developed different classes of antibodies which were specific for at least one viral protein. N- and S-specific IgA dictated the early development of the antibody response in a disease severity-dependent manner. This finding is in accordance with previous reports highlighting that circulating IgA contributed to a greater extent to the antibody response against SARS-CoV-2 compared to IgG also in biological fluids other than serum or plasma, i.e. saliva and bronchoalveolar lavages (BAL) (20, 80–82). Interestingly, the frequency of IgA-expressing plasmablasts was higher than cells expressing IgG early upon infection and these cells showed a phenotype consistent with those found at mucosal sites (80). The magnitude of the IgA response we found in plasma from the infected subjects was greater than that of IgG, probably as a consequence of the hyperinflammatory environment observed in critically ill patients. Indeed, the exuberant IgA production may result from increased levels of TGF-β and IL-10 during SARS-CoV-2 infection, which promote antibody switching (83, 84). IgA response in saliva declined more rapidly than systemic IgA after infection (85). The evidences on the role of the humoral immune response in the resolution of COVID-19 are conflicting. While we and others (86–88) have detected higher antibody titers in patients with moderate/severe symptoms than in asymptomatic or mild patients, other studies found the opposite, raising the question whether antibody levels determine the outcome of the disease (79). The IgA-mediated FcαRI signaling on myeloid cells during an infection with IgA-opsonized pathogens induces proinflammatory responses (89). However, having virus-specific antibodies does not always imply the establishment of a protective immunity, since pathogens may have evolved escape mechanisms, such as epitope masking. Quite surprisingly, we observed higher neutralization ability in patients affected by moderate to severe symptoms compared to mild patients, and both IgA and IgG isotypes contributed to the functional activity of patients’ plasma. Studies pointed out to the importance of considering thresholds of antibody concentration and neutralization activity as correlate of protection from SARS-CoV-2 infection in both non-vaccinated and vaccinated subjects (20, 90). Moreover, in addition to neutralization, other functional profiles may emerge during natural infection and may move the needle to determine the outcome of the disease. Indeed, Fc-effector functions and specific IgA and IgG subclasses were strongly linked with the ability to rapidly clear viremia, thus promoting a better COVID-19 outcome (91). In severe COVID-19 patients, mucosal non-neutralizing SARS-CoV-2 IgA persisted after viral clearance in the lung, suggesting that IgA may exert harmful functions beside protective neutralization at mucosal level (82). Interestingly, anti-S IgG immune complexes promoted the alveolar macrophages polarization towards a pro-inflammatory phenotype only in severe patients due to the low fucosylation of the antibody Fc tail (92). However, the evaluation of the humoral immunity alone is not sufficient to assess the ability of the host to control the viral infection. SARS-CoV-2 specific humoral and T-cell responses did not correlate in a cohort of vaccinated subjects evaluated at different timepoints, indicating that assays evaluating T-cell responses could provide complementary information to those inferred by serological tests (93). In the same cohort of patients, extensively characterized by flow cytometry, we also assessed the frequency of SARS-CoV-2 specific T cells at different timepoints after hospital admission. Specific T-cell responses are elicited in 1–2 weeks from antigen exposure. Samples at T0 (during the acute phase of the disease) were collected at a median of 8 (IQR 5–12) days from symptom onset (**Table 1**). Therefore, in order to avoid false negative responses in this timeframe, we decided to evaluate SARS-CoV-2 specific T-cell responses selectively during the follow-up period. Searching for an assay that concurrently had a low detection limit, was not dependent on predefined HLA alleles, and allowed the assessment of both CD4 and CD8 immunity, we gave preference to IFN-γ ELISpot assay. PBMC were stimulated with both SARS-CoV-2 Spike and Nucleocapsid peptide pools, which were added to the same well. The PepTivators employed are pools of overlapping peptides, consisting mainly of 15-mer sequences, specifically designed to stimulate T cells reactive to either the Spike or the Nucleocapsid protein of the SARS-CoV-2 Wuhan wild-type strain.

We observed that poor T-cell mediated immune responses to SARS-CoV-2 epitopes were correlated with the severity of the disease. T-cell functional defects were profound in the severe group since even the ability to release IFN-γ upon polyclonal stimulation was impaired. This may be related to the high expression of exhaustion markers on CD4^+^ T cells observed in patients-specific metaclusters. In line with the model proposed by Crotty & Sette (27), SARS-CoV-2 specific T-cell response was greatly delayed and appeared only at 6 months after the infection in survivors belonging to the severe group.

One of the most prominent alteration in the T-cell phenotype herein described is the abnormal accumulation of late differentiated effector CD4^+^ and CD8^+^ T cells in COVID-19 patients. The latter were found to be highly informative in discriminating the patients’ outcome when challenged in the multiparametric analysis, being one of the variables selected by the decision tree. These cells were characterized by the low expression of costimulatory receptors and molecules involved in the homing to the lymph node, which may result in defective CD8 priming and stimulation toward new antigens (94, 95). The Naïve-Memory imbalance, together with TCR repertoire reduction, T-cell senescence and dysfunction are hallmarks of T-cell aging that actively contributes to the development of Inflammaging (96). Such condition involves several defects in the immune response mounted toward pathogens leading to an increased release of inflammatory cytokines and mediators, to myeloid cells dysfunction, to an accumulation of senescent cells and ultimately to tissue deterioration.

In our study, the occurrence of SARS-CoV-2 reinfection was notably rare, with only one patient experiencing reinfection in October 2020, prior to receiving the first vaccine dose in May 2021. This patient had amounts of T and B cell subsets as well as monocytes and NK cells within the interquartile ranges of the entire cohort. Therefore, we would not consider this patient as having an abnormal immune response. However, this isolated case of reinfection within our cohort limits our ability to conclusively determine if the biological perturbations observed conferred protective immunity against SARS-CoV-2 reinfection. Furthermore, it is essential to investigate how vaccination may influence cellular and humoral immune signatures. Studies with larger sample sizes, encompassing diverse populations and extended follow-up periods, are needed to better understand the implications of immunological changes post-infection and their potential role in mediating protection against reinfection, as well as to explore the full impact of vaccination on these immune dynamics.

In summary, our data show that the characterization of both innate and adaptive immune cell subsets is needed to accurately predict the broad variability of clinical manifestations of SARS-CoV-2 infection upon hospital admission. The perturbation of myeloid, NK, B and T-cell compartments persists several months beyond the acute phase of the disease and is possibly involved in the development of late-emerging symptoms. Furthermore, Bregs could be associated to the different susceptibility of developing long COVID, probably due to their function in the maintenance of tolerance, by limiting persistence of systemic inflammation and incidence of auto-immune diseases. A significant reduction of the immunosuppressive Breg compartment, along with an expansion of Beff cells, has been described by other studies in severe COVID-19 patients (77). Interestingly, this peculiar B cell dynamics was also observed in autoimmune pathologies characterized by chronic inflammation (97), while the resolution of lung pathology in COVID-19 patients was found to be associated with higher proportions of IL-10^+^ B cells, suggesting that these cells could be important in suppressing excess inflammation and positive long-term outcomes (77).

## 4 Conclusion

In conclusion, patients with a lower frequency of NK-cell precursors and CD14^+^CD91^+^ monocytes, and a higher frequency of CD8+ Effector Memory T cells at hospital admission had a lower probability to survive. Moreover, the lack of regulatory B cells at one month after discharge was associated with an increased risk of developing long COVID at six months. We believe that these data are relevant for the early identification of patients at risk of developing severe and persistent symptoms upon SARS-CoV-2 infection, offering a novel tool for individualized approaches aimed at monitoring and treating COVID-19 patients.

## 5 Methods

### 5.1 Patients and study design

Adult patients who were admitted to the emergency department during the first wave of COVID-19 pandemic were included in the study. Patients were classified according to a 6-point ordinal scale ranging from those who met discharged criteria for at least 72 hours (score=1) to those who succumbed the disease (score=6), as previously described(Yeming 32). Next, the 6 scores were further clustered into three categories: “Mild” for a score of 1–2-3, “Moderate” for a score of 4, and “Severe” for a score of 5–6, based on the worst disease manifestations during the whole course of the disease.

### 5.2 Sample collection and inclusion criteria

Peripheral blood samples were collected from patients who tested positive for SARS-CoV-2 at hospital admission. Longitudinal samples were also harvested during follow-up visits, at 1 (T1M), 3 (T3M) and 6 (T6M) months after hospital discharge. The median time from symptom onset was 43 days for T1M (IQR 33–55), 99 days for T3M (IQR 91–117) and 193 days for T6M (IQR 181–223). Peripheral Blood Mononuclear cells (PBMC) were obtained from whole blood by density gradient centrifugation (Lymphoprep, Sentinel diagnostics) and frozen for subsequent analysis. Plasma, serum and swabs samples were also frozen and stocked in the institutional biobank, the Biological Resource Center (CRB-OSR) (Num ID CRB in BBMRI-ERIC: bbmri-eric:ID: IT_1383758011993577). Inclusion criteria required patients to be over 18 years of age, have a SARS-CoV-2 positive swab and the availability of at least 10^7^ frozen PBMC at hospital admission. Samples were excluded if PBMC viability was below 50% after thawing, as assessed by trypan blue exclusion or staining with viability dyes. Age matched healthy donor (HD) samples collected before the pandemic were also analyzed.

### 5.3 Study approval

All patients received treatment according to Institutional programs, and written informed consent was obtained for the use of medical records and immunological studies. The study, named “ImmCOVID”, was conducted within a non-interventional study and received approval from San Raffaele Institutional Ethical Committee on April 20, 2020.

### 5.4 Polychromatic flow cytometry of monocytes

PBMC were immediately washed after thawing and fixed in PBS-BSA buffer with Thrombofix according to manufacturer’s indications. After an overnight incubation at 4°C, cells were labelled with a cocktail of fluorochrome-conjugated antibodies listed in **Supplementary Table 2** and acquired within 4 hours with a Navios cytometer (Beckman Coulter). Data were analyzed using FCS express Flow Cytometry Software (*De Novo* Software).

### 5.5 Polychromatic flow cytometry of NK cells

Frozen PBMCs were thawed at 37°C in water bath with a gentle swirling, rapidly transferred in a 15 ml tube and spun at 160g for 10 minutes. The supernatant was removed and cells were resuspended in 10 ml of culture medium, placed in a 5% C0_2_ incubator and let to rest for 2 hours at 37°C. Cells were then centrifuged (160g for 10 minutes), the supernatant was removed and incubated with LIVE/DEAD fixable Aqua dead cell stain (0.125ul/100µl sample; life technology) for 30 minutes at 4°C. After washing with FACS Buffer (PBS supplemented with 1% Fetal Bovine Serum; FBS), cells were incubated with a mix containing all the fluorochrome-conjugated antibodies described in **Supplementary Table 3** for 30 minutes at 4°C. After washing, samples were acquired (≥250’000 events for each sample) with a Navios cytometer (Beckman Coulter) and analyzed using FlowJo version10.8.0 (BD Biosciences). Live lymphocytes were gated on a live/dead area versus side scatter area pseudo-color dot plot and NK-cells were identified starting from CD3^-^ lymphocytes, as described in **Supplementary Figure 4**.

### 5.6 Polychromatic flow cytometry of B cells

At hospital admission and at 1 and 3 months after discharge, PBMC samples were thawed and kept in culture in RPMI (Lonza) supplemented with 10% FBS, Glutamine (1%) and Penicillin/Streptomycin (1%). After 16 hours culture PBMC were stained with viability dye (**Supplementary Table 4**), and, after washing, cells were incubated with 2.5 μg Fc Blocker (BD Biosciences cat. 564220) in 20 μl PBS for 15 minutes at room temperature (RT) in the dark. Titrated antibodies listed in **Supplementary Table 4** were mixed in 30 μl of BD Horizon Brilliant Blue Buffer (BD Bioscences) and incubated with the cells for 20 minutes at RT in the dark. After 3 washings the cells were fixed and acquired with FACSymphony A5 flow cytometer (BD Biosciences) with instrumental setting as described in (98).

### 5.7 Polychromatic flow cytometry of T cells

At hospital admission and at 1 and 3 months after discharge, PBMC samples were thawed and cultured in RPMI (Lonza) supplemented with 10% FBS, Glutamine (1%) and Penicillin/Streptomycin (1%). After an overnight culture, Fixable Viability Dye 620 (BD Biosciences) was added at a concentration of 1:1000 for 10 minutes at RT. Next, PBMC were washed and stained with the fluorochrome-conjugated antibodies included in **Supplementary Table 5** for 20 minutes at RT. All washing steps were performed with autoMacs Rinsing Solution (Miltenyi) supplemented with 0.5% Bovine Serum Albumin (Miltenyi). After staining, cells were fixed using a fixation buffer (Biolegend) and acquired within 24 hours using a FACSymphony A5 cytometer (BD Biosciences). Data were analyzed by FlowJo software version 10 (BD Biosciences).

### 5.8 High dimensional analysis of T cells

After compensation optimization, exclusion of doublets and dead cells and lymphocyte selection based on physical parameters, the CD3^+^ events were exported to a new.fcs file for each sample. The application CytoChain (52) was used for sequential dataset optimization, dimensionality reduction analysis and clustering. First, the dataset was downsided to contain approximately 1.3 million events CD3^+^ T cells. Next, data were cleaned using the flowAI package to select the most stable flow rate, ensuring in-range signal acquisition and dynamic range stability. All the channels’ fluorescence intensities values were transformed with the arcSinh function (cofactor 150). After that, the spade package was used to remove outliers, accounting for 0–3% of the events from each sample. This process aimed to improve the clustering performance and enhance the quality of the resulting map after dimensionality reduction. The sample’s size was further reduced to balance the number of events in each disease and severity condition.

The optimized flowset underwent dimensionality reduction analysis by t-distributed Stochastic Neighbour Embedding (t-SNE, perplexity 30) and clustering. The Flow-Self Organized Map (FlowSOM) algorithm identified 100 clusters, which were then collapsed into 25 metaclusters according to phenotype similarities by the ConsensusClusterPlus package. Moreover, a heatmap was generated reporting the ratio of fluorescence intensity with respect to the maximum for each metacluster. The optimized flowset was also analyzed by FlowJo version 10 (BD Biosciences). Relevant signatures were backtracked in the original samples by manual gating to validate cytoChain results.

### 5.9 Cytokine and chemokines quantification

Plasma-EDTA was obtained by centrifugation of venous blood, immediately frozen and maintained at -80°C until subsequent analyses. The concentration in the plasma of CXCL10 was measured using a commercial ELISA kit (DY266 R&D DuoSet ELISA Development System). Multiplex immunoassays (Bio-Rad) based on Luminex technology were used for the quantification of 17 biomarkers among cytokines and chemokines in human samples (G-CSF, GM-CSF, IFN-γ, IL-1β, IL-2, IL-4, IL-5, IL-6, IL-7, IL-8, IL-10, IL-12 (p70), IL-13, IL-17°, MCP-1 (MCAF), MIP-1β, TNF-α), according to the manufacturer’s instructions (Bio-Plex Pro™ Human Cytokine 17-plex). Data were measured on a Bio-Plex 200 System and calculated using Bio-Plex Manager 6.0 and 6.1 software.

### 5.10 IFN-γ ELISpot for SARS-CoV-2 specific antigens

Frequencies of IFN-γ-producing SARS-CoV-2-specific T cells were evaluated by enzyme-linked immunospot assay (ELISpot) using cryopreserved PBMC harvested at follow-up timepoints. PBMC were thawed and cultured for two hours in IMDM (Lonza) supplemented with 10% Human Serum, Glutamine (1%) and Penicillin/Streptomycin (1%) and low doses of recombinant human IL-2 (rhIL-2, 20 UI/ml, Novartis). The PBMC were then washed to eliminate rhIL-2. A high quality ELISpot kit (Mabtech) was used featuring pre-coated plates and one-step detection to increase the reproducibility. Briefly, 400’000 PBMC were seeded per well (99) and stimulated for 16–20 hours with two libraries of overlapping peptides spanning both the spike and the nucleocapsid proteins: PepTivator SARS-CoV-2 S and N (1 μg of each peptide per ml, Miltenyi). Anti-CD3 monoclonal antibody was used as positive control, and irrelevant peptides as negative control. In case of stimulations with SARS-CoV-2 peptide libraries and irrelevant peptides, cells were pulsed for 1 hour at 37°C before the overnight stimulation. In each condition an anti-CD28 monoclonal antibody (1 μg/ml, BD Biosciences) was added to provide costimulatory signal and increase T-cell costimulation. Spot forming cells were quantified by the ImmunoCapture 7.0 software (TLC ELISpot Reader). Negative controls (i.e. unstimulated T cells and/or T cells stimulated with the irrelevant peptides) were subtracted. Results were expressed as specific spot forming cells (SFC)/400’000 PBMC. Alternative functional assays used to measure specific T-cell responses require a higher number of PBMC (1x10^6^ PBMC per well (29) versus 0.4x10^6^ PBMC per well required for the ELISpot assay (99) or the knowledge of patients’ HLA alleles (i.e. multimers), which were unknown in our cohort.

### 5.11 Cells for lentiviral-pseudotyped particles production and neutralization assay

Human embryonic kidney 293 cells (HEK 293T/17 cells) were acquired from Programme EVA Centre for AIDS Reagents, National Institute for Biological Standards and Control (NIBSC, UK) and cultured in Dulbecco’s modified Eagle’s medium supplemented with 4.5 mg/ml glucose, 2 mML-glutamine (Lonza), 100 units/ml penicillin-streptomycin (Lonza) and 10% of FBS (Euroclone). The cells were incubated at 37°C, 5% CO_2_ in humidified atmosphere.

### 5.12 Detection of SARS-CoV-2-specific antibodies

IgA, IgG, and IgM specific to the S1 and S2 subunits were detected in plasma samples as previously described using an in-house ELISA (20). Briefly, 0.1 μg/well of recombinant S1 or S2 protein (Abeomics) was plated at on Maxisorp 96-well plates (Thermo Scientific) and incubated overnight at 4°C in 50 mM carbonate/bicarbonate buffer at pH 9.5. After blocking for 1 h at 37°C with PBS containing 10% BSA and 0.05% Tween 20, duplicate of 1:100 diluted plasma samples were added and incubated for 1 h at 37°C. The plates were then treated for 30 min at 37°C with 1:6,000 horseradish peroxidase (HRP)-conjugated goat antihuman IgA, antihuman IgG, or anti-human IgM (Southern Biotech). Plates were developed using TMB 2C (KPL-SeraCare), and the reaction was stopped with 10% sulfuric acid after 5 minutes. The optical density (OD) values were measured at wavelengths of 450 and 620 nm using a PowerWave ELISA reader (BioTek). If OD values were higher than 2.0, samples were further diluted and retested. Plasma samples from 40 healthy donors collected before COVID-19 pandemic served as negative controls. The cutoff value was determined by calculating the mean OD plus three times the standard deviation obtained from healthy individuals.

IgG, IgA, and IgM to the NP were measured through SARS-CoV- 2 (COVID-19) ELISA Kits (Novatec) following the manufacturer’s instruction. An initial screening for NP reactivity was performed to identify positive candidate control samples for S1- and S2- binding assays. Cutoff values were arbitrarily established at 10 Arbitrary Units (AU) for both the in-house ELISA and Novatec ELISA kits, with specificity ranging from 98.26% to 100%. AU values were calculated with the formula: “[mean OD (sample)×10 AU (cutoff)]/mean OD (cutoff)” and then converted to Binding Arbitrary Units for ml (BAU/ml), according to the formula: “AU×1000”.

The WHO International Standard (WHO IS, National Institute for Biological Standards and Control, NIBSC, UK, cod. 20/136) and the WHO Reference Panel (WHO RP, NIBSC, cod. 20/268) for anti-SARS-CoV-2 antibody were tested at 1:100 dilution in the in-house ELISA for S1 and S2 and the Novatec kit for NP as described above. This was done to confirm concordance with our results.

### 5.13 Purification of IgG and IgA

HiTrap Protein G HP 1 ml column (GE Healthcare) and Peptide M-Agarose 2 ml column (InvivoGen Europe) were used to purify IgG and IgA, respectively. These columns were used to process 300 μl of plasma following the manufacturing instructions and an automatic HPLC system (Biologic DuoFlow, Bio-Rad Laboratories) was utilized for this purification process.

### 5.14 Production, titration and neutralization of SARS-CoV-2 lentiviral-pseudotyped particles

HEK293 T/17 at the 60% of confluence were co-transfected with the pcDNA 3.1 plasmid bearing the full-length spike of ancestral SARS-CoV-2, the HIV gag-pol plasmid and the firefly luciferase-expressing plasmid pCSFLW using FuGENE^®^ HD Transfection Reagent (Promega) in accordance with the manufacturer’s instructions. After transfection, supernatant containing the pseudotyped viruses was harvested 72h post−transfection, centrifuged at 500xg for 5 minutes to remove cellular debris and filtered with a 0.45-mm filter. Aliquots of the collected pseudotyped viruses were stored at - 80°C.

For titration of pseudovirus stocks and the neutralization assay, HEK 293T/17 cells were transfected with pACE2 and pTMPRSS2 plasmids for 24 h. Virus infectivity was determined by titration on HEK 293T/17-ACE2/TMPRSS2 cells as previously described (100).

Plasma samples were heat-inactivated by incubation at 56°C for 30 minutes. Neutralization assays were performed by incubating 10^6^ relative light units RLU of pseudotyped viruses with endpoint twofold serial dilutions of heat-inactivated plasma samples or plasma purified IgG and IgA. The incubation was done at 37°C, 5% CO_2_ for 1 hour before addition of 10^4^ HEK 293T/17-ACE2/TMPRSS2 cells per well. After 72 h at 37°C, the cells were lysed using a Luciferase Assay (Promega) and luciferase activity was measured using a Victor luminometer (PerkinElmer). The neutralization titers were expressed as IC50 values, defined as the concentration of plasma required to achieve half-maximal neutralization.

### 5.15 Statistics

Unpaired tTest or Mann-Withney tests were carried out to compare two datasets. Comparisons between three or more groups were performed either by ordinary one-way Anova or Kruskal-Wallis test. The Shapiro-Wilk test was employed to check for normality. In the case of two independent variables, the comparison between three or more groups was performed by two-way Anova followed by Tukey’s multiple comparison test. Graph-pad prism 9 software was used for such statistical analysis.

To identify clinical, laboratory and immunological parameters that best discriminate patients with different disease severity, a classification tree model was estimated (rpart R package). Due to the high number of variables considered, a variable selection procedure based on Random Forest was applied to obtain a first reduction of the number of variables, before estimating the classification tree model. While the classification tree method handles the presence of missing data through the usage of surrogate splits, the Random Forest algorithm needs the imputation of missing data for using the entire dataset. Therefore, the variable selection procedure consisted in: (1) imputing 50 times the missing data; (2) estimating a Random Forest and applying the Boruta algorithm for variable selection (101) on each complete dataset (randomForest and Boruta R packages); (3) considering for successive analysis only variables selected in at least 50% of the times. After the variable selection step, the classification tree algorithm was applied to the entire dataset through the use of surrogate splits for the estimation of the model.

Kaplan–Meier estimator was used to estimate overall survival curves and logrank test was employed for comparing survival curves among groups.

Spearman’s correlations analysis between variables selected by the decision tree and all variables at T0 was performed. False discovery rate (FDR) correction was applied to account for multiple testing.

The classification tree estimation, the survival analysis and the correlation analysis were performed with R 4.2.1 (https://www.rproject.org/).

## Data availability statement

The raw data supporting the conclusions of this article will be made available by the authors, without undue reservation.

## Ethics statement

The studies involving humans were approved by San Raffaele Institutional Ethical Committee. The studies were conducted in accordance with the local legislation and institutional requirements. The participants provided their written informed consent to participate in this study.

## Author contributions

MN: Conceptualization, Data curation, Investigation, Methodology, Supervision, Writing – original draft, Writing – review & editing. RD: Conceptualization, Data curation, Investigation, Methodology, Supervision, Writing – review & editing. RCh: Conceptualization, Data curation, Investigation, Methodology, Writing – original draft. NM: Conceptualization, Data curation, Investigation, Methodology, Writing – original draft. CDe: Conceptualization, Data curation, Investigation, Methodology, Writing – original draft. GSi: Conceptualization, Data curation, Investigation, Methodology, Writing – original draft. NL: Conceptualization, Data curation, Investigation, Methodology, Writing – original draft. PRa: Data curation, Methodology, Software, Writing – review & editing. FCu: Data curation, Methodology, Software, Writing – review & editing. ET: Methodology, Supervision, Writing – review & editing. SD: Investigation, Writing – review & editing. DA: Formal analysis, Software, Writing – review & editing. VB: Investigation, Methodology, Writing – review & editing. ER: Supervision, Writing – review & editing. FM: Methodology, Software, Writing – review & editing. AMe: Data curation, Writing – review & editing. EC: Data curation, Writing – review & editing. CT: Data curation, Writing – review & editing. CP: Investigation, Methodology, Writing – review & editing. RCa: Investigation, Methodology, Writing – review & editing. FSi: Investigation, Methodology, Writing – review & editing. IM: Investigation, Methodology, Writing – review & editing. FSa: Investigation, Methodology, Writing – review & editing. SG: Investigation, Methodology, Writing – review & editing. VL: Supervision, Writing – review & editing. EV: Supervision, Writing – review & editing. PC: Supervision, Writing – review & editing. AMa: Supervision, Writing – review & editing. GSc: Supervision, Writing – review & editing. PD: Supervision, Writing – review & editing. LL: Supervision, Writing – review & editing. CDi: Supervision, Writing – review & editing. MM: Supervision, Writing – review & editing. FCi: Conceptualization, Funding acquisition, Supervision, Writing – review & editing. PRo: Conceptualization, Supervision, Writing – review & editing. CB: Conceptualization, Funding acquisition, Supervision, Writing – original draft, Writing – review & editing.
