# Supplementary material for: The longitudinal characterization of immune responses in COVID-19 patients reveals novel prognostic signatures for disease severity, patients’ survival and long COVID
Source: Front Immunol. 2024 Jul 29;15:1381091. doi: 10.3389/fimmu.2024.1381091 (PMC11317765; doi:10.3389/fimmu.2024.1381091)
Supplement: Supplementary file 1 [file DataSheet_1.docx]

Supplementary Material

Supplementary Material for

**The longitudinal characterization of immune responses in COVID-19 patients reveals novel prognostic signatures for disease severity, patients’ survival and long COVID**

- Supplementary Table 1. Additional patients characteristics
- Supplementary Table 2. Flow cytometry reagents and antibodies for the monocytes panel
- Supplementary Table 3. Flow cytometry reagents and antibodies for NK-cell panels
- Supplementary Table 4. Flow cytometry reagents and antibodies for the B-cell panel
- Supplementary Table 5. Flow cytometry reagents and antibodies for the T-cell panel
- Supplementary Table 6. Variables at T0 included in a classification tree analysis for the classification of disease severity
- Supplementary Table 7. Variables at T0 included in a classification tree analysis for long COVID incidence
- Supplementary Table 8. Long COVID symptoms
- Supplementary Table 9. Variables at T1M included in a classification tree analysis for long COVID incidence
- Supplementary Figure 1. Classification of disease scores in “Mild”, “Moderate” and “Severe” categories was associated to biomarkers of disease severity
- Supplementary Figure 2. Gating strategy and representative plots for the monocyte panel
- Supplementary Figure 3. IL-6, MIP-1b, IL-8 and MCP-1 plasma levels at hospital admission.
- Supplementary Figure 4. Gating strategy for NK cell subset classification.
- Supplementary Figure 5. Unclassical CD16^dim^ effector NK cells.
- Supplementary Figure 6. Gating strategy for the B-cell panel.
- Supplementary Figure 7. Patients affected by COVID-19 display a lower proportion of immunomodulatory T cells (Tregs) compared to healthy donors
- Supplementary Figure 8. High dimensional analysis of CD3+ cells by cytochain
- Supplementary Figure 9. Gating Strategies for the validation of relevant phenotypic signatures on CD3 cells from COVID-19 patients.
- Supplementary Figure 10. Association between the variables selected by the decision tree for the classification of disease severity and all the input variables at T0

**Supplementary Table 1. Additional patient characteristics**

Abbreviations: CAD, coronary artery disease; T1D, type 1 diabetes; T2D, type 2 diabetes; CKD, chronic kidney disease; COPD, chronic obstructive pulmonary disease, TBC, tuberculosis, HIV, human immunodeficiency virus.

**Supplementary Table 2. Flow cytometry reagents and antibodies for the monocytes panel**

| **Specificity** | **Fluorochrome** | **Clone** | **Company** | **Catalogue number** |
| --- | --- | --- | --- | --- |
| CD14 | PE-Cy7 | RMO52 | Beckman Coulter | A22331 |
| CD16 | PE-Cy5 | 3G8 | Biolegend | 302010 |
| CD86 | APC | IT2.2 | Biolegend | 305412 |
| CD91 | PE | A2MR-α2 | Becton Dickinson | 550497 |
| HLA-DR | APC-Cy7 | L243 | Biolegend | 307618 |
| compensatory beads | NA | NA | Beckman coulter | 552843 |
| Thrombofix ® | NA | NA | Beckman coulter | 6607130 |

**Supplementary Table 3. Flow cytometry reagents and antibodies for NK-cell panels**

| **Specificity** | **Fluorochrome** | **Clone** | **Company** | **Catalogue number** |
| --- | --- | --- | --- | --- |
| CD3 | AF-700 | SP-34 | Becton Dickinson | 557917 |
| CD8 | PE-Cy7 | RPA-T8 | BD-Pharmingen | 560917 |
| CD56 | PE-CF594 | NCAM 16.2 | Becton Dickinson | 564849 |
| CD16 | APC-H7 | 3G8 | Becton Dickinson | 560195 |
| CD69 | PE-Cy5.5 | CH/4 | Thermo | MHCD6918 |
| CD159a (NKG2A) | APC | REA110 | Miltenyi | 130-113-563 |
| CD159c (NKG2C) | Vio Bright-FITC | REA 205 | Miltenyi | 130-117-707 |
| CD158 e1 (3DL1) | BV421 | NKB1 | Becton Dickinson | 742979 |
| CD158 e1/e2 (3DL1/DS1) | PE-Vio770 | REA 168 | Miltenyi | 130-104-486 |
| CD158 a/h (2DL1/DS1) | Vio-Blue | 11PB6 | Miltenyi | 130-125-246 |
| CD158 a/g (2DL1/DS5) | APC | 143211 | R&D System | FAB1844A |
| CD158 h (2DS1) | AF488 | 1127B | R&D System | FAB8887G |
| CD158b1/b2/i KIR2DL2/3/DS2/4 | PE | 180704 | R&D System | FAB1848P |

**Supplementary Table 4. Flow cytometry reagents and antibodies for the B-cell panel**

| **Specificity** | **Fluorochrome** | **Clone** | **Company** | **Catalogue number** |
| --- | --- | --- | --- | --- |
| CD86 | AF488 | IT2.2 | Biolegend | 305414 |
| CD11c | PerCP-Cy5.5 | B-ly6 | Becton Dickinson | 565227 |
| CD95 | APC | DX2 | Biolegend | 305612 |
| CD3 | APC-H7 | SK7 | Becton Dickinson | 641415 |
| CD19 | APC-Cy7 | HIB19 | Biolegend | 302218 |
| CXCR5 | BV421 | J252D4 | Biolegend | 356920 |
| CD24 | BV510 | ML5 | Biolegend | 311126 |
| IgM | BV605 | MHM-88 | Biolegend | 314524 |
| CD80 | BV650 | L307.4 | Becton Dickinson | 564158 |
| CD27 | BV711 | O323 | Biolegend | 302834 |
| CD21 | BV750 | B-ly4 | Becton Dickinson | 747254 |
| CD25 | BV786 | BC96 | Biolegend | 302638 |
| IgG | BUV395 | G18-145 | Becton Dickinson | 564229 |
| Viability dye | BUV496 | NA | Invitrogen | L23105 |
| CD40 | BUV563 | 5C3 | Becton Dickinson | 741381 |
| CD69 | BUV737 | FN50 | Becton Dickinson | 612817 |
| IgD | BUV805 | IA6-2 | Becton Dickinson | 742039 |
| CD1c | PE | L161 | Biolegend | 331506 |
| CXCR3 | PE-Dazzle | G025H7 | Biolegend | 353736 |
| CD138 | PE-Cy5.5 | B-A38 | Beckman coulter | B96786 |
| CD38 | PE-Cy7 | HB-7 | Biolegend | 356608 |

**Supplementary Table 5. Flow cytometry reagents and antibodies for the T-cell panel**

| **Specificity** | **Fluorochrome** | **Clone** | **Company** | **Catalogue number** |
| --- | --- | --- | --- | --- |
| CD3 | BUV661 | UCHT1 | Becton Dickinson | 612964 |
| CD4 | BUV496 | SK3 | Becton Dickinson | 564651 |
| CD8 | APC-H7 | SK1 | Becton Dickinson | 560179 |
| CD25 | BV421 | M-A251 | Becton Dickinson | 562442 |
| CD26 | APC | FR10-11G9 | Miltenyi | 130-120-699 |
| CD27 | BV605 | L128 | Becton Dickinson | 562655 |
| CD28 | BV650 | CD28.2 | Becton Dickinson | 740593 |
| CD38 | BB515 | HIT2 | Becton Dickinson | 564498 |
| CD45RA | AlexaFluor700 | HI100 | Becton Dickinson | 560673 |
| CD62L | BUV805 | DREG-56 | Becton Dickinson | 742024 |
| CD95 | PE-Cy7 | DX2 | Becton Dickinson | 561636 |
| CD127 | BB700 | HIL-7RM21 | Becton Dickinson | 566398 |
| CD137 (4-1BB) | BUV737 | 4B4-1 | Becton Dickinson | 741861 |
| CD279 (PD-1) | BV480 | EH12.1 | Becton Dickinson | 566112 |
| CD366 (TIM-3) | BV711 | 344823 | Becton Dickinson | 747959 |
| CXCR5 | BUV395 | RF8B2 | Becton Dickinson | 740266 |
| HLA-DR | BUV563 | G46-6 | Becton Dickinson | 748340 |
| ICOS | PE | DX29 | Becton Dickinson | 557802 |
| KLRG1 | BV786 | 2F1/KLRG1 | Biolegend | 138429 |
| Viability dye (FVS620) | PE CF594 | NA | Becton Dickinson | 564996 |

**Supplementary Table 6. Variables at T0 included in a classification tree analysis for the classification of disease severity**

T0 (n=62)

|  | **n** | **mean** | **sd** | **median** | **min** | **max** | **Q0.25** | **Q0.75** |
| --- | --- | --- | --- | --- | --- | --- | --- | --- |
| P/F_admission | 60 | 228.33 | 105.77 | 254.76 | 2.97 | 398.57 | 154.08 | 305.6 |
| P/F_nadir | 60 | 188.1 | 111.65 | 157 | 41 | 398.57 | 81.88 | 290.32 |
| BodyTemperature | 56 | 37.6 | 1.07 | 37.5 | 36 | 40 | 36.8 | 38.5 |
| Hemoglobin | 62 | 13.94 | 1.54 | 13.95 | 9.5 | 16.8 | 13.2 | 15 |
| ALC | 62 | 1.34 | 0.82 | 1.2 | 0.3 | 5.7 | 0.83 | 1.48 |
| AST | 62 | 56.71 | 36.47 | 47.5 | 16 | 269 | 35.25 | 62.5 |
| ALT | 62 | 48.98 | 29.64 | 40 | 13 | 153 | 28.25 | 55.5 |
| LDH | 60 | 427.67 | 183.63 | 400 | 145.2 | 1101 | 323.75 | 468.75 |
| PCR | 62 | 113.9 | 92.88 | 108.2 | 1.8 | 332 | 34.18 | 167.7 |
| Creatinine | 62 | 0.98 | 0.3 | 0.94 | 0.47 | 1.76 | 0.75 | 1.15 |
| Viral_Load | 44 | 5440146.44 | 34233382.72 | 1689.23 | 0 | 227298178.57 | 228.51 | 92497.46 |
| S1-IgA | 44 | 23959.35 | 31285.23 | 10604.14 | 1414.68 | 141684 | 4653.91 | 27297.04 |
| S2-IgA | 44 | 14915.28 | 28659.22 | 5553.75 | 783.29 | 174166.67 | 3127.43 | 15605.39 |
| NP-IgA | 43 | 102934.81 | 205388.2 | 28796.99 | 580.14 | 1204142.58 | 9530 | 98255.64 |
| S1-IgG | 44 | 12136.58 | 9164.44 | 8457.2 | 1627.36 | 30745.05 | 4135.33 | 19989.26 |
| S2-IgG | 44 | 12062.25 | 5975.13 | 12930.47 | 1460 | 22963.99 | 8110.06 | 15489.98 |
| NP-IgG | 44 | 23528.78 | 19627.45 | 21601.86 | 2600.73 | 82720.66 | 6459.42 | 30145.63 |
| S1-IgM | 44 | 12977.17 | 9723.78 | 11460.06 | 468.75 | 45327.12 | 6377.57 | 16034.18 |
| S2-IgM | 44 | 7191.65 | 5019.21 | 6053.61 | 369.49 | 25179.6 | 3541.73 | 9282.5 |
| IC50 | 44 | 7948.43 | 23113.3 | 848.33 | 71.7 | 117166.47 | 83.85 | 4742.5 |
| ID50 | 53 | 35249.63 | 227019.95 | 563 | 10 | 1656017 | 92 | 5163 |
| RBD_IgG | 55 | 26.42 | 52.37 | 5.08 | 0 | 266.21 | 0.61 | 21.52 |
| RBD_IgM | 55 | 9.69 | 12.2 | 5.9 | 0.44 | 70.6 | 3.15 | 10.95 |
| RBD_IgA | 55 | 5.62 | 9.85 | 2.3 | 0.39 | 57.37 | 1.33 | 5.12 |
| S2_IgG | 40 | 792.05 | 2751.44 | 38.7 | 0.01 | 16028.03 | 2.74 | 275.17 |
| S1S2_IgG | 54 | 53.42 | 65.12 | 28.65 | 0.07 | 298.39 | 5.17 | 76.34 |
| S1S2_IgM | 54 | 13.03 | 23.03 | 7.23 | 0.54 | 167.22 | 4.26 | 12.73 |
| S1S2_IgA | 54 | 12.89 | 14.04 | 7.76 | 0.49 | 73.69 | 3.38 | 18.77 |
| NP_IgG | 53 | 21.37 | 21.04 | 12.22 | 0.24 | 72.01 | 3.05 | 38.76 |
| CD3 T cells | 59 | 67.29 | 17.72 | 71.14 | 12.97 | 92.37 | 58.36 | 80.57 |
| CD4 T cells | 59 | 63.94 | 15.62 | 63.93 | 12.16 | 89.27 | 53.11 | 77.28 |
| CD8 T cells | 59 | 24.46 | 12.54 | 22.76 | 4.08 | 57.37 | 14.39 | 33.24 |
| Early memory CD4+ T cells (mc#1) | 59 | 21.56 | 9.03 | 21.03 | 3.18 | 45.58 | 14.55 | 26.51 |
| Early memory CD8+ T cells (mc#10) | 59 | 3.72 | 4.57 | 1.98 | 0 | 18.55 | 0.77 | 3.92 |
| Exhausted effector memory CD4+ T cells (mc#11) | 59 | 1 | 1.52 | 0.48 | 0 | 9.52 | 0.19 | 1.21 |
| CD28+ CD127+ Memory CD8+ T cells (mc#12) | 59 | 11.19 | 6.95 | 9.71 | 0.41 | 37.67 | 6.4 | 14.75 |
| CD28- CD127- memory CD4+ T cells (mc#17) | 59 | 11.54 | 8.09 | 10.37 | 1.62 | 40.64 | 5.77 | 14.7 |
| Effector memory CD8+ T cells (mc#13) | 59 | 42.47 | 15.92 | 40.68 | 9.78 | 83.42 | 28.7 | 54.46 |
| Activated effector memory CD8+ T cells (mc#13) | 59 | 4.17 | 4.21 | 2.65 | 0 | 20.75 | 1.73 | 4.66 |
| cTfh | 59 | 6.86 | 3.86 | 6.29 | 1.38 | 24.3 | 4.27 | 8.51 |
| Tregs | 59 | 1.64 | 1.3 | 1.12 | 0 | 6.41 | 0.77 | 2.62 |
| CD14+CD16+ | 33 | 6.25 | 6.44 | 3.59 | 0.37 | 31.09 | 1.82 | 7.61 |
| CD14+CD86+ | 33 | 19.66 | 15.13 | 15.03 | 2.24 | 61.46 | 7.52 | 27.51 |
| CD14+CD91+ | 33 | 47.94 | 21.07 | 49.29 | 2.17 | 83.06 | 38.35 | 63.35 |
| CD14+HLADR+ | 33 | 28.23 | 13.04 | 27.09 | 1.44 | 61.91 | 21.16 | 35 |
| Classical Monocytes | 32 | 63.16 | 26.73 | 73.5 | 8.09 | 92.5 | 46.58 | 85.38 |
| Intermediate Monocytes | 32 | 2.99 | 3.32 | 1.77 | 0.1 | 15.1 | 1.02 | 3.05 |
| Non-classical Monocytes | 32 | 29.89 | 26.75 | 18.03 | 0.8 | 88.41 | 7.73 | 47.9 |
| CXCL10 (pg/ml) | 52 | 689.8 | 575.68 | 656.33 | 15.62 | 2376.59 | 152.74 | 1062.43 |
| Naive B cells | 56 | 63.29 | 22.41 | 68.55 | 0.04 | 91.7 | 59.15 | 77.78 |
| USM B cells | 56 | 4.09 | 4.73 | 2.25 | 0.01 | 21.3 | 1.33 | 4.49 |
| Memory B cells | 56 | 12.22 | 11.35 | 8.4 | 0.8 | 51.8 | 5.45 | 14.32 |
| DNMemory B cells | 56 | 19.05 | 14.75 | 15.5 | 2.84 | 87.9 | 10.82 | 23.55 |
| Plasmablasts | 56 | 0.63 | 0.98 | 0.2 | 0 | 4.13 | 0.05 | 0.62 |
| Breg | 56 | 1.81 | 1.79 | 1.29 | 0 | 9.73 | 0.56 | 2.66 |
| Exhausted NK cells | 55 | 28.09 | 10.84 | 27.9 | 6.87 | 50.45 | 20.65 | 36.8 |
| NK cell precursors | 55 | 1.95 | 1.65 | 1.47 | 0.1 | 7.68 | 0.72 | 2.69 |
| Effector CD16^dim^ NK cells | 55 | 21.19 | 7.91 | 21.44 | 7.02 | 45.35 | 14.8 | 25.5 |
| Effector CD16^+^ NK cells | 55 | 26.71 | 12.75 | 24.45 | 5.46 | 60.5 | 17.6 | 35.5 |
| Memory-like NK cells | 55 | 3.51 | 6.12 | 1.85 | 0.04 | 31.15 | 0.69 | 3.08 |
| Exhausted NK cells_NKG2A CD69+ | 55 | 8.01 | 7.1 | 5.79 | 0.27 | 32.7 | 2.73 | 11.65 |
| Exhausted NK cells_NKG2C CD69+ | 55 | 4.7 | 4.62 | 2.87 | 0 | 16 | 1.35 | 6.57 |
| Effector CD16+ NK cells_NKG2A CD69+ | 55 | 14.52 | 12.26 | 12.2 | 0.02 | 60.7 | 5.02 | 19.6 |
| Effector CD16+ NK cells_NKG2C CD69+ | 55 | 10.18 | 12.08 | 6.83 | 0.1 | 59.1 | 1.71 | 13.3 |

**Supplementary Table 7. Variables at T0 included in a classification tree analysis for long COVID incidence**

LONG COVID Physical 6 months (n=34)

No 21 (61.8%)

Yes 13 (38.2%)

| **Variables at T0 (n=34)** | **n** | **mean** | **sd** | **median** | **min** | **max** | **Q0.25** | **Q0.75** |
| --- | --- | --- | --- | --- | --- | --- | --- | --- |
| P/F_admission | 32 | 247.29 | 96.03 | 263.81 | 36 | 398.57 | 200.89 | 305.6 |
| P/F_nadir | 32 | 207.12 | 101.82 | 230.24 | 48 | 398.57 | 120.25 | 295 |
| BodyTemperature | 29 | 37.69 | 1.08 | 37.7 | 36 | 39.6 | 36.8 | 38.5 |
| Hemoglobin | 34 | 13.81 | 1.68 | 13.85 | 9.5 | 16.4 | 12.9 | 14.93 |
| ALC | 34 | 1.16 | 0.53 | 1.1 | 0.3 | 3.2 | 0.8 | 1.3 |
| AST | 34 | 58.82 | 45.15 | 46 | 16 | 269 | 35 | 62.5 |
| ALT | 34 | 50.5 | 33.16 | 41 | 13 | 153 | 25.25 | 63.25 |
| LDH | 32 | 419.28 | 163.41 | 399.5 | 185 | 1086 | 324.75 | 452.25 |
| PCR | 34 | 109 | 94.51 | 74.75 | 3.9 | 332 | 31 | 151.55 |
| Creatinin | 34 | 0.92 | 0.27 | 0.84 | 0.49 | 1.53 | 0.73 | 1.1 |
| Viral_Load | 26 | 75488 | 234221.7 | 639.82 | 0 | 1168015 | 0 | 11239 |
| S1-IgA | 30 | 28769.51 | 36176.08 | 12305.06 | 1414.68 | 141684 | 4551.3 | 39871.91 |
| S2-IgA | 30 | 17597.49 | 33962.63 | 6879.03 | 783.29 | 174166.7 | 3922.5 | 13469.95 |
| NP-IgA | 30 | 123317.6 | 238671.3 | 28897.88 | 580.14 | 1204143 | 14306.95 | 157877.8 |
| S1-IgG | 30 | 14207.15 | 9261.91 | 15366.78 | 1627.36 | 30745.05 | 5402.14 | 21837.03 |
| S2-IgG | 30 | 12899.68 | 5380.59 | 13327.56 | 1978.02 | 22963.99 | 8944.99 | 15925.6 |
| NP-IgG | 30 | 25423.5 | 20354.44 | 23127.62 | 4246.15 | 82720.66 | 8064.94 | 29866.71 |
| S1-IgM | 30 | 13650.82 | 10753.41 | 12175.24 | 468.75 | 45327.12 | 5393.94 | 16965.85 |
| S2-IgM | 30 | 7525.66 | 5576.14 | 5680 | 369.49 | 25179.6 | 3445.18 | 9340.5 |
| IC50 | 30 | 10080.02 | 27649 | 948.88 | 71.7 | 117166.5 | 71.74 | 5187.5 |
| ID50 | 30 | 57173.03 | 302008.7 | 543 | 10 | 1656017 | 95 | 2001.5 |
| RBD_IgG | 31 | 16.75 | 24.74 | 6.7 | 0.02 | 88.04 | 0.64 | 16.9 |
| RBD_IgM | 31 | 10.72 | 13.95 | 5.92 | 0.45 | 70.6 | 2.76 | 12.62 |
| RBD_IgA | 31 | 3.22 | 2.82 | 2.34 | 0.41 | 11.77 | 1.54 | 3.6 |
| S2_IgG | 26 | 161.08 | 326.62 | 20.57 | 0.01 | 1497.09 | 1.99 | 209.14 |
| S1S2_IgG | 30 | 48.15 | 54.79 | 27.34 | 0.07 | 173.58 | 4.89 | 68.12 |
| S1S2_IgM | 30 | 15.29 | 30.22 | 7.23 | 1.59 | 167.22 | 3.61 | 12.35 |
| S1S2_IgA | 30 | 12.79 | 10.97 | 8.38 | 0.49 | 42.89 | 5.36 | 19.64 |
| NP_IgG | 30 | 17.16 | 17.97 | 9.81 | 0.24 | 57.18 | 3.06 | 33.91 |
| CD3 T cells | 31 | 68.47 | 18.22 | 75.16 | 12.97 | 90.11 | 56.81 | 82.39 |
| CD4 T cells | 31 | 62.76 | 17.48 | 63.37 | 12.16 | 89 | 54.94 | 75.97 |
| CD8 T cells | 31 | 25.96 | 13.05 | 25.32 | 4.08 | 57.37 | 14.82 | 33.38 |
| Early memory CD4+ T cells (mc#1) | 31 | 21.31 | 9.57 | 18.69 | 3.18 | 40.36 | 14 | 25.94 |
| Early memory CD8+ T cells (mc#10) | 31 | 4 | 5.13 | 1.81 | 0.19 | 18.55 | 1 | 4.3 |
| Exhausted effector memory CD4+ T cells (mc#11) | 31 | 0.82 | 0.95 | 0.43 | 0 | 4.07 | 0.18 | 1.1 |
| CD28+ CD127+ Memory CD8+ T cells (mc#12) | 31 | 11.42 | 7.49 | 9.71 | 0.41 | 37.67 | 7.2 | 13.54 |
| CD28- CD127- memory CD4+ T cells (mc#17) | 31 | 13.36 | 9.13 | 11.57 | 2.11 | 40.64 | 8 | 16.3 |
| Effector memory CD8+ T cells (mc#13) | 31 | 39.75 | 14.13 | 38.32 | 11.39 | 63.25 | 27.31 | 49.52 |
| Activated effector memory CD8+ T cells (mc#13) | 31 | 3.73 | 3.38 | 2.65 | 0.55 | 14.23 | 1.82 | 4.13 |
| cTfh | 31 | 6.6 | 2.83 | 7.12 | 2.26 | 13.2 | 4.1 | 8.18 |
| Tregs | 31 | 1.74 | 1.48 | 1.11 | 0 | 6.41 | 0.67 | 2.62 |
| CD14+CD16+ | 19 | 4.9 | 4.76 | 3.24 | 0.37 | 17 | 1.59 | 6.45 |
| CD14+CD86+ | 19 | 21.6 | 17.26 | 16.68 | 2.24 | 61.46 | 7.78 | 30.16 |
| CD14+CD91+ | 19 | 49.04 | 21.5 | 51.53 | 2.17 | 83.06 | 39.84 | 64.61 |
| CD14+HLADR+ | 19 | 29.37 | 14.26 | 26.94 | 1.44 | 61.91 | 22.12 | 35 |
| Classical Monocytes | 18 | 69.46 | 22.19 | 73.5 | 8.09 | 92.5 | 55.21 | 88.51 |
| Intermediate Monocytes | 18 | 2.01 | 1.99 | 1.42 | 0.1 | 7.18 | 0.81 | 2.51 |
| Non-classical Monocytes | 18 | 24.22 | 22.85 | 17.88 | 4.1 | 88.41 | 6.44 | 37.5 |
| CXCL10 (pg/ml) | 27 | 642.28 | 556.15 | 663.83 | 15.62 | 1792.25 | 126.56 | 1012.18 |
| Naive B cells | 29 | 61.6 | 25.08 | 67.1 | 0.9 | 91.7 | 60.1 | 76 |
| USM B cells | 29 | 3.92 | 3.35 | 2.68 | 0.25 | 14.3 | 2.02 | 4.76 |
| Memory B cells | 29 | 14.76 | 13.59 | 8.7 | 0.8 | 51.8 | 5.88 | 17.6 |
| DNMemory B cells | 29 | 18.45 | 12.14 | 13.5 | 2.84 | 58.2 | 11.3 | 25.2 |
| Plasmablasts | 29 | 0.66 | 0.99 | 0.19 | 0 | 4.13 | 0.07 | 0.62 |
| Breg | 29 | 1.41 | 1.34 | 0.93 | 0 | 5.26 | 0.52 | 2.03 |
| Exhausted NK cells | 30 | 28.84 | 9.53 | 28.17 | 13.9 | 46.05 | 22.29 | 36.8 |
| NK cell precursors | 30 | 2.27 | 1.71 | 1.98 | 0.1 | 5.73 | 0.74 | 3.94 |
| Effector CD16^dim^ NK cells | 30 | 18.79 | 7.02 | 19.2 | 7.02 | 41.9 | 13.31 | 22.6 |
| Effector CD16^+^ NK cells | 30 | 25.52 | 11.91 | 22.58 | 5.46 | 49.4 | 17.95 | 33.48 |
| Memory-like NK cells | 30 | 4.5 | 7.87 | 2.42 | 0.04 | 31.15 | 0.42 | 3.63 |
| Exhausted NK cells_NKG2A CD69+ | 30 | 7.39 | 6.38 | 5.67 | 0.99 | 28.7 | 2.69 | 9.75 |
| Exhausted NK cells_NKG2C CD69+ | 30 | 5.08 | 4.52 | 3.14 | 0.13 | 16 | 1.67 | 8.12 |
| Effector CD16+ NK cells_NKG2A CD69+ | 30 | 13.72 | 11.5 | 10.97 | 0.9 | 43.9 | 4.47 | 17.58 |
| Effector CD16+ NK cells_NKG2C CD69+ | 30 | 7.33 | 6.43 | 5.28 | 0.3 | 24.7 | 2.46 | 12.12 |

**Supplementary Table 8. Long COVID symptoms**

| **Long COVID symptoms** | **Patients, n (%)** |
| --- | --- |
| Systemic (smell or taste alterations, arthralgias, myalgias, or fatigue) | 4 (18.2) |
| Respiratory (dyspnea, cough, or chest-pain) | 14 (63.6) |
| Neurologic (brain fog, headache, or insomnia) | 2 (9.1) |
| Psychiatric (anxiety or post- traumatic stress disorder) | 8 (36.4) |

**Supplementary Table 9. Variables at T1M included in a classification tree analysis for long COVID incidence**

| **Variables at T1M (n=33)** | **n** | **mean** | **sd** | **median** | **min** | **max** | **Q0.25** | **Q0.75** |
| --- | --- | --- | --- | --- | --- | --- | --- | --- |
| S1-IgA | 29 | 34638.1 | 31128.68 | 22100 | 3925.75 | 129193 | 17190 | 37480 |
| S2-IgA | 29 | 10082.85 | 5241.86 | 8820 | 1253.26 | 21460 | 6084.1 | 13911.4 |
| NP-IgA | 29 | 55736.05 | 84707.9 | 29323.31 | 2690 | 364218.8 | 15360 | 51810 |
| S1-IgG | 29 | 19918.98 | 4414.34 | 21780 | 5070.75 | 23389.91 | 20047.17 | 22311.32 |
| S2-IgG | 29 | 18320.21 | 5323.6 | 17179.49 | 5558.61 | 31358.56 | 14926.74 | 20010 |
| NP-IgG | 29 | 42590.95 | 14349.93 | 46420 | 7076.92 | 64400 | 33170 | 52338.46 |
| S1-IgM | 29 | 22207.4 | 18931.66 | 14290 | 357.14 | 58996.2 | 6040 | 42170 |
| S2-IgM | 29 | 12048.55 | 11196.49 | 8720 | 417.16 | 47946.79 | 4020 | 17238.73 |
| IC50 | 29 | 9558.94 | 13293.72 | 4587.93 | 699.12 | 45170.07 | 2222.22 | 9520.1 |
| ID50 | 28 | 4400.02 | 5967.8 | 2931 | 10 | 31886 | 1380.5 | 4536 |
| RBD_IgG | 28 | 3864.48 | 6074.1 | 1371.28 | 0.12 | 28141.57 | 561.54 | 4474.91 |
| RBD_IgM | 28 | 9.9 | 12.49 | 4.08 | 0.39 | 45.1 | 1.62 | 13.77 |
| RBD_IgA | 28 | 8.18 | 9.1 | 4.44 | 0.37 | 41.64 | 2.96 | 10.11 |
| S2_IgG | 28 | 2484.29 | 6423.46 | 922 | 0.03 | 34560.17 | 239.74 | 2237.92 |
| S1S2_IgG | 19 | 10398.56 | 9789.47 | 6608.45 | 1.72 | 32961.62 | 3850.07 | 17090.93 |
| S1S2_IgM | 7 | 49.06 | 106.34 | 7.44 | 5.1 | 290.05 | 7 | 13.41 |
| S1S2_IgA | 7 | 12.14 | 14.12 | 6.61 | 1.37 | 39.92 | 2.87 | 15.67 |
| NP_IgG | 7 | 42.68 | 22.59 | 40.44 | 3.02 | 69.66 | 33.89 | 58.94 |
| CD3 T cells | 28 | 68.97 | 18.89 | 74.65 | 26.74 | 96.98 | 59.61 | 81.58 |
| CD4 T cells | 28 | 55.26 | 17.43 | 57.8 | 18.39 | 94.3 | 44.2 | 65.75 |
| CD8 T cells | 28 | 36.35 | 16.21 | 33.78 | 3.86 | 70.84 | 26.63 | 44.94 |
| Early memory CD4+ T cells (mc#1) | 28 | 23.67 | 11.17 | 22.06 | 5.94 | 56.65 | 18.06 | 29.2 |
| Early memory CD8+ T cells (mc#10) | 29 | 2.84 | 2.81 | 1.87 | 0.07 | 10.67 | 0.57 | 4.04 |
| Exhausted effector memory CD4+ T cells (mc#11) | 28 | 0.37 | 0.48 | 0.16 | 0.01 | 2.16 | 0.09 | 0.51 |
| CD28+ CD127+ Memory CD8+ T cells (mc#12) | 29 | 12.62 | 8.39 | 11.23 | 1.21 | 43.13 | 7.59 | 14.2 |
| CD28- CD127- memory CD4+ T cells (mc#17) | 29 | 6.98 | 6.61 | 4.64 | 1.04 | 32.26 | 3.36 | 8.9 |
| Effector memory CD8+ T cells (mc#13) | 28 | 43.24 | 20.87 | 38.5 | 7.52 | 84.52 | 25.94 | 59.45 |
| Activated effector memory CD8+ T cells (mc#13) | 28 | 4.44 | 3.9 | 3.23 | 0.28 | 15.2 | 2.09 | 6.06 |
| cTfh | 28 | 8.29 | 2.59 | 8.31 | 3.69 | 13.12 | 6.7 | 10.14 |
| Tregs | 28 | 2.73 | 1.33 | 2.6 | 0.67 | 5.4 | 1.78 | 3.39 |
| CD14+CD16+ | 14 | 3.55 | 2.56 | 3.02 | 0.8 | 7.86 | 1.55 | 5.33 |
| CD14+CD86+ | 14 | 22.3 | 13.09 | 22.41 | 4.08 | 41.54 | 11.25 | 31.18 |
| CD14+CD91+ | 14 | 56.47 | 13.62 | 58.51 | 35.27 | 75.83 | 44.46 | 67.68 |
| CD14+HLADR+ | 14 | 38.27 | 10.77 | 34.45 | 25.22 | 56.38 | 29.66 | 49.14 |
| Classical Monocytes | 13 | 61.6 | 15.09 | 63.91 | 37 | 81.26 | 53.24 | 72.15 |
| Intermediate Monocytes | 13 | 2.09 | 2 | 1.16 | 0.25 | 5.74 | 0.47 | 3.83 |
| Non-classical Monocytes | 13 | 27.78 | 15.52 | 27.75 | 6.12 | 54.56 | 13.86 | 35.76 |
| CXCL10 (pg/ml) | 31 | 147.53 | 348.92 | 15.62 | 15.62 | 1924.7 | 15.62 | 158.19 |
| Naive B cells | 27 | 60.72 | 26.13 | 67.7 | 2.26 | 89.1 | 45.75 | 81.7 |
| USM B cells | 27 | 7.15 | 6.67 | 5 | 0.11 | 30.9 | 3.68 | 8.53 |
| Memory B cells | 27 | 17.95 | 15.64 | 14.3 | 1.69 | 54 | 5.81 | 24.35 |
| DNMemory B cells | 27 | 13.18 | 8.56 | 10.6 | 3.69 | 40 | 7.5 | 13.95 |
| Plasmablasts | 27 | 0.24 | 0.39 | 0.13 | 0 | 1.92 | 0.06 | 0.22 |
| Breg | 27 | 1.52 | 1.63 | 1.17 | 0 | 5.6 | 0.1 | 2.43 |
| Exhausted NK cells | 17 | 25.33 | 17.11 | 21.35 | 6.71 | 66.95 | 14.38 | 27.85 |
| NK cell precursors | 17 | 2.69 | 1.99 | 2.07 | 0.06 | 7.34 | 1.48 | 3.38 |
| Effector CD16^dim^ NK cells | 17 | 17.87 | 8.36 | 17.1 | 5.89 | 37.77 | 11.45 | 22.45 |
| Effector CD16^+^ NK cells | 17 | 31.94 | 15.02 | 30.25 | 3.45 | 61.3 | 26 | 37 |
| Memory-like NK cells | 17 | 5.38 | 5.24 | 4.72 | 0.33 | 15.4 | 0.75 | 7.67 |
| Exhausted NK cells_NKG2A CD69+ | 17 | 7.89 | 7.34 | 4.46 | 0.3 | 22.5 | 2.82 | 16.4 |
| Exhausted NK cells_NKG2C CD69+ | 17 | 3.84 | 3.45 | 3.06 | 0.1 | 11.9 | 1 | 4.47 |
| Effector CD16+ NK cells_NKG2A CD69+ | 17 | 17.08 | 14.54 | 8.79 | 2.4 | 45.4 | 6.49 | 27.9 |
| Effector CD16+ NK cells_NKG2C CD69+ | 17 | 10.34 | 9.49 | 7.1 | 0.13 | 27.9 | 1.24 | 18.5 |

**Supplementary Figure 1. Classification of disease scores in “Mild”, “Moderate” and “Severe” categories was associated to biomarkers of disease severity.**

The lowest value of the oxygenation index (Nadir PaO_2_/FiO_2_) (**A**), Absolute Lymphocyte Counts (ALC, **B**), C-Reactive Protein (CPR, **C**) and lactate dehydrogenase (LDH, **D**) values at hospital admission are shown for the three disease categories, “Mild” for a score of 1-2-3, “Moderate” for a score of 4 and “Severe” for a score of 5-6. Min to max values are shown. Statistical analyses were performed by Kruskal-Wallis test (multiple comparisons).

**Supplementary Figure 2. Gating strategy and representative plots for the monocyte panel**

Monocytes were identified within the CD14+ population. Classical (CD14++CD16-), intermediate (CD14++CD16+) and non-classical (CD14+CD16++) monocytes as well as the expression of CD86, CD91 and HLA-DR were also identified within CD14+ population. (**A**) Representative dot plots of a mild patient at admission and one and three months after discharge. (**B**) correlation between the expression of CD91 on monocytes and the concentration of the chemokine CXCL10.


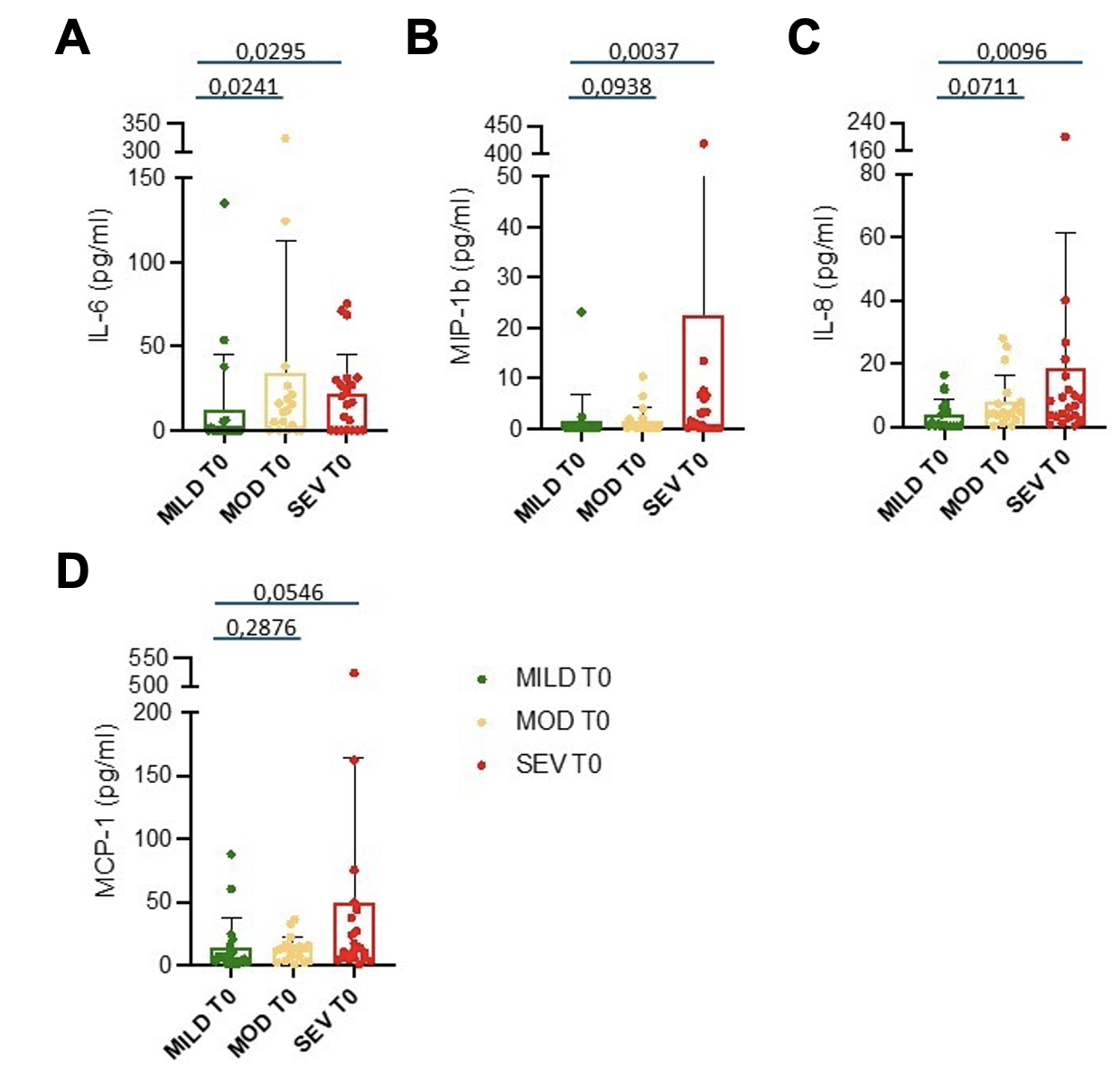


**Supplementary Figure 3**. **IL-6, MIP-1b, IL-8 and MCP-1 plasma levels at hospital admission.** Concentration of IL-6 (**A**), MIP-1b (**B**), IL-8 (**C**), and MCP-1 (**D**) in Mild, Moderate (MOD) and Severe (SEV) COVID-19 patients at hospital admission (T0).

**Supplementary Figure 4. Gating strategy for NK cell subset classification.** Gating strategy used to identify NK subpopulations from single, alive, CD3^-^ blood cells. Flow cytometry dot plot of CD56 versus CD16 of representative healthy donor (bottom-left) shows the main NK cell subsets: **(1)** CD56^dim^/CD16^-^ **[exhausted NK cells]**; **(2)** CD56^brigh^t/CD16^-^ **[NK cell precursors]**; (3) CD56^bright^/CD16^+^; (**4**) CD56^dim^/CD16^dim^ **[effector CD16^dim^ NK cells]**; **(5)** CD56^dim^/CD16^brigth^ **[effector CD16^+^ NK cells]**; **(6)** CD56^-^/CD16^bright^ **[memory-like NK cells]**; (7) CD56^-^/CD16^dim^.

**Supplementary Figure 5. Unclassical CD16^dim^ effector NK cells.** (**A**) Percentage of effector CD16^dim^ NK cells and of (**C**) CD69+ effector CD16^dim^ NK cells in healthy donor (HD) and Mild, Moderate (MOD) and Severe (SEV) COVID-19 patients, at hospital admission (T0). Longitudinal evaluation of the percentage of (**B**) effector CD16^dim^ NK cells and (**D**) CD69+ effector CD16^dim^ NK cells in Mild, Moderate and Severe COVID-19 patients, at the indicated timepoints: hospital admission (T0), 1 month (T1M) and 3 months (T3M) after discharge. Statistical significance is calculated by Kurskal-Wallis’s test, following Dunn correction for multiple comparisons. * p<0.05; ** p<0.01; *** p<0.001.

**Supplementary Figure 6. Gating strategy for the B-cell panel. A.** Gating strategy: CD19+ CD3- B cells were identified among lymphocytes after exclusion of dead cells and doublets as indicated. **B.** Phenotype of double negative memory (DNM) B-cell subset in healthy donors and COVID-19 patients at hospital admission (T0). DNM B cells from each subject were in silico grouped and 4 different concatenates were obtained each for healthy donors, mild, moderate and severe patient cohorts. The expression profile of markers is shown. Vertical bars indicate the negative threshold

**Supplementary Figure 7. Patients affected by COVID-19 display a lower proportion of immunomodulatory T cells (Tregs) compared to healthy donors**

T-cell phenotypic characterization of COVID-19 patients compared to age- and sex-matched healthy donors (manual gating). Relative proportion of CD3 (**A**), and of CD4 (**B**) and CD8 (**C**) T cell subsets are shown for Mild, Moderate and Severe patients, at hospital admission (T0). **D**. CD4^+^CD127^low^CD25^bright^ regulatory T cells (Tregs) are shown for representative HD and COVID-19 patient gated on CD3^+^CD4^+^. Relative proportion of Tregs are shown for Mild, Moderate and Severe patients and compared to HD at T0 (**E**) and 1 month (T1M, **F**) and 3 months (T3M, **G**) after discharge. Mean with Standard deviation are shown. Statistical analyses were performed by ordinary one-way ANOVA (multiple comparisons).

**Supplementary Figure 8. High dimensional analysis of CD3+ cells by cytochain. A.** PCA analysis**. B.** scaled fluorescence distribution of the indicated markers in the t-SNE map (marker map). **C.** heatmap for 25 metaclusters; the ratio of fluorescence intensity of each marker with respect to the maximum is reported as in the color-legend. Green and red rectangles highlight HD and COVID-19-specific clusters, respectively. Unsupervised high dimensional analysis and clusterization of CD3+ events were performed by cytoChain. The PCA analysis, markers maps and the Heatmap were generated by cytoChain. **D** and **E**. Validation of the phenotypic signatures associated to HD-specific or patient-specific metaclusters by manual gating. Mean with Standard deviation are shown. Statistical analysis in D and E were performed by ordinary one-way ANOVA (multiple comparisons).

**Supplementary Figure 9. Gating Strategies for the validation of relevant phenotypic signatures on CD3 cells from COVID-19 patients.**

**A**. Gating Strategy of the relative proportion of “CD62L+CD95+CD127+KLRG1-CD28+CD56- cells” on CD3+CD4+ T cells (metacluster #1). **B** relative proportion of CXCR5+ circulating follicular helper T cells (cTfh) on CD3+CD4+ T cells (metacluster #1). **C**. Relative proportion of “CD45RA+CD62L+CD95-CD127+KLRG1-CD28+CD56- cells” on CD3+CD8+ T cells (metacluster #10). **D.** Relative proportion of “CD45RA-CD127+CD28+ cells” on CD3+CD8+ T cells (metacluster #12). **E.** Relative proportion of “CD62L-CD28-PD1+HLA-DR- cells” (metacluster #11; right) and of “CD62L-CD28-PD1-KLRG1- cells” (metacluster #17; left) on CD3+CD4+ T cells. **F.** Relative proportion of “CD62L-CD28-CD56- cells” (left) and of “CD62L-CD28-CD56-CD38+HLADR+ cells” (right) on CD3+CD8+ T cells (metacluster #13).


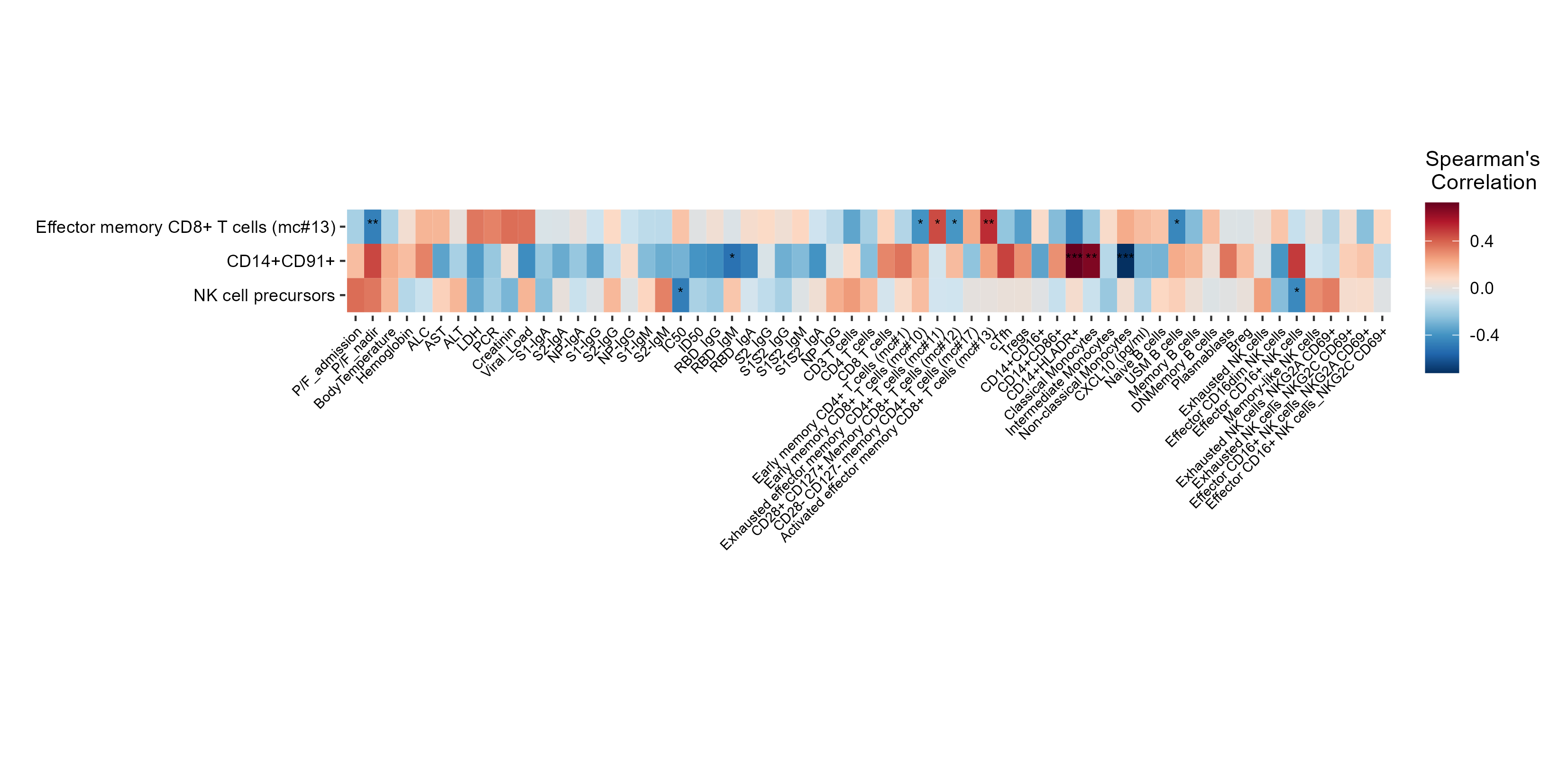


**Supplementary figure 10. Association between the variables selected by the decision tree and all the input variables at T0.** Spearman’s correlations between variables selected by the decision tree and all the variable at T0. The magnitude of each correlation is denoted with a color, whereby the red color indicates a positive correlation and blue color represents a negative correlation, such that the deeper the color, the stronger is the correlation. Levels of statistical significance with False Discovery Rate (FDR) correction are denoted as: * p<0.05, ** p<0.01, *** p<0.001.
